# HIF-mediated hierarchical hypoxic adaptation, a novel paradigm in heart failure pathogenesis: is there a role for targeted therapies?

**DOI:** 10.3389/fphar.2026.1771529

**Published:** 2026-03-16

**Authors:** Lyu Yongjie, Wei Jingjing, Su Yimeng, Wang Aolong, Yu Rui, Wang Jianru, Li Bin, Wang Yongxia, Wang Xinlu, Zhu Mingjun

**Affiliations:** 1 Heart Center, The First Affiliated Hospital of Henan University of Chinese Medicine, Zhengzhou, China; 2 Henan Evidence-Based Medicine Center of Chinese Medicine, The First Affiliated Hospital of Henan University of Chinese Medicine, Zhengzhou, China; 3 Collaborative Innovation Center of Prevention and Treatment of Major Diseases by Chinese and Western Medicine, Henan University of Chinese Medicine, Zhengzhou, China

**Keywords:** cardioprotection, heart failure, hypoxia, hypoxia-inducible factors, traditional Chinese medicine

## Abstract

Heart failure (HF) is a terminal cardiovascular syndrome related to systemic hypoxia. Hypoxia is considered a fundamental pathophysiological process, and the resulting tissue response depends on the severity and duration of exposure. Hypoxia-inducible factors (HIFs) promote adaptation to hypoxic conditions by regulating the expression of multiple hypoxia-responsive genes. Its short-term activation during acute hypoxia exerts cardioprotective effects, whereas chronic activation induces pathological hypertrophy, depending on the disease context. Therefore, HIF-mediated hypoxic responses in HF may involve hierarchical adaptations to hypoxia. This review discusses the role of HIFs in the physiology and pathology of HF, focusing on metabolic remodeling, angiogenesis, cardiac inflammation, and circadian influences, as well as their potential effects on myocardial performance. Furthermore, the therapeutic potential of HIF-targeting compounds in HF treatment will be reviewed. Overall, whether targeting HIF-induced changes in HF is an effective strategy remains to be established; thus, research in this field is urgently needed.

## 1 Introduction

Heart failure (HF), a global epidemic characterized by substantial morbidity and mortality, affects over 64 million people worldwide (Khan et al., 2024; Savarese et al., 2023). In China, an estimated 12.1 million adults have HF, with the rapidly ageing population and increasing cardiovascular disease risk factors posing a severe challenge to public health and socioeconomic stability (Wang et al., 2021). According to data from the China Cardiovascular Association Registry and national insurance databases, the projected prevalence of HF in China will increase from 13.1 million in 2021 to 22.7 million in 2035, representing a 73% growth (Wang H. et al., 2025). Both European and Chinese guidelines recommend guideline-directed medical therapy (GDMT) for HF (MCDonagh et al., 2021; Heidenreich et al., 2022; MCDonagh et al., 2023; Chinese Society of Cardiology, 2025). However, even when patients receive GDMT treatment, risks of HF deterioration and death still exist, underscoring the urgent need for deeper investigation into the pathophysiological process of HF and effective HF therapy targets (Butt et al., 2024).

Emerging evidence suggests that HF is a complex syndrome with heterogeneity in pathophysiology. After initial injuries such as myocardial ischemia and hypoxia, myocardial contractility declines. As a result, compensatory mechanisms are activated, including neuroendocrine activation, ventricular remodeling, and metabolic remodeling. This maladaptive process ultimately leads to pump failure and an energetic crisis (Parmley, 1989; Kumar et al., 2019; Hinton et al., 2024; Thorp and Filipp, 2025; Andreadou et al., 2025). The crucial role of HIFs as master regulators linking hypoxia to pathological changes in HF is well established. Although HIFs have been reported to induce hypoxia-responsive genes and restore cellular oxygen homeostasis, there remains considerable uncertainty about their potential protective or detrimental role in HF progression. Previous studies have revealed that HIFs exhibit a preferential binding bias toward HIF-1α over HIF-2α, which may contribute to their dual effects on cellular processes (Schödel et al., 2011). Sustained HIF-1α signaling promotes oxidative stress, inflammation, and fibrosis, whereas HIF-2α is believed to counteract these effects. Consequently, imbalances in the interactions between HIF-1α and HIF-2α could exacerbate chronic HF (Packer, 2020). In contrast, HIF-3α serves as a negative regulator of both HIF-1α and HIF-2α. However, our understanding of its mechanisms remains limited. To date, no unified theory or comprehensive study has fully elucidated the overall impact of HIFs in HF. This review systematically summarizes recent advances in the HIF system, explores its multifaceted roles in HF, and highlights potential therapeutic agents targeting the HIF pathway to improve HF outcomes. Ultimately, it seeks to offer theoretical foundations for establishing HIF as a viable therapeutic target in HF.

## 2 Stability of HIFs under normoxia

Purification by DNA affinity chromatography revealed that HIF-1 is a heterodimer consisting of α and β subunits (Wang et al., 1995). In mammals, the α subunits comprise HIF-1, HIF-2, and HIF-3, while the β subunit is also known as the aryl hydrocarbon receptor nuclear translocation protein (ARNT) (Sousa et al., 2019). Under normoxic conditions, the stability of HIF-1α and HIF-2α proteins is negatively regulated by prolyl hydroxylase domain protein (PHD)-dependent hydroxylation. This hydroxylation leads to von Hippel–Lindau protein (VHL)-dependent ubiquitination and subsequent proteasomal degradation (Semenza, 2014a). The oxygen-dependent asparaginyl hydroxylase factor inhibiting HIF-1 (FIH-1) is also a key regulator of the HIFs’ C-terminal transactivation domain. This enzyme provides a direct link between oxygen sensing and HIF-mediated transcription (Lando et al., 2002; Lisy and Peet, 2008). Collectively, this class of oxygen-dependent enzymes comprises critical regulatory components of the hypoxic response pathway.

### 2.1 Functions of HIF-1α in normoxia and pseudohypoxia

HIF-1α mediates cellular responses to oxygen deprivation. A gene-expression array has demonstrated that more than 1,000 genes are directly transactivated by HIF-1α (Schödel et al., 2011; Xia et al., 2009). Two major categories of proteins encoded by HIF-1α target genes are those that enhance oxygen delivery and those that reduce oxygen consumption (Semenza, 2014b). However, it can also maintain stability under normoxic conditions, such as in pseudohypoxia. For instance, WD repeat domain 5 (WDR5) serves as a key regulator that enhances HIF-1α activity in cholangiocarcinoma. HIF-1α acts as a critical effector in WDR5-driven metastasis, even under normoxic conditions (95% air and 5% CO_2_), without hypoxic induction (Chen et al., 2021). Similarly, experimental evidence indicates that the ROS-mediated pseudohypoxic state may trigger HIF-1α activation (Mialet-Perez and Belaidi, 2024). The ROS-mediated pseudohypoxia promotes a self-reinforcing loop between NOX enzymes and HIF-1α, sustaining fibrosis progression. Under ambient air conditions (∼18% pericellular O_2_), TGF-β1 activates NOX2 and upregulates NOX4, generating ROS that oxidize the Fe^2+^ cofactor of PHD2 and impair HIF-1α hydroxylation (Choi et al., 2025). Moreover, a recent study has shown that early mitochondrial ROS signaling, mediated by the mitochondrial Na^+^/Ca^2+^ exchanger (NCLX), is essential for HIF activation. Specifically, inhibiting or knocking down NCLX prevents hypoxia from stabilizing HIF-1α and HIF-2α proteins or inducing downstream gene transcription. However, in mouse embryonic fibroblasts (MEFs) with naturally elevated basal ROS levels, hypoxia activates HIFs even after NCLX knockout. Conversely, reducing these basal ROS levels restores NCLX dependency for HIF activation (Choya-Foces et al., 2024). These findings support the role of HIF-1α in adaptive and pathological processes, extending beyond hypoxia to include cancer metabolism and ROS-mediated pseudohypoxic states.

### 2.2 HIF-2α is a driver of peroxisome

HIF-2α exhibits high sequence homology with HIF-1α (Dengler et al., 2014). It shares with HIF-1α the transcriptional activation of specific target genes, such as vascular endothelial growth factor A (VEGFA) and glucose transporter 1 (GLUT1) (Sousa et al., 2019). However, the functions of HIF-2α and HIF-1α do not overlap. Transcriptome and proteome analyses reveal that HIF-1α is linked to glycolysis, while HIF-2α is mainly associated with lipid metabolism (Hoefflin et al., 2020). Numerous studies have demonstrated the regulatory role of HIF-2α signaling in peroxisome function (Walter et al., 2014; Schönenberger et al., 2015). A noteworthy study in liver-specific VHL/HIF-2α knockout mice suggested that HIF-2α-mediated pexophagy degrades the autophagy receptor NBR1, which localizes to peroxisomes, thereby altering lipid composition (Walter et al., 2014). It was also reported that peroxisome abundance is reduced in VHL-deficient human clear cell renal cell carcinomas with high HIF-2α levels (Walter et al., 2014). Another study in ARNT-deficient mice with cardiomyopathy found that ARNT directly regulates peroxisome proliferator-activated receptor alpha (PPARα) expression by binding to its promoter and forming a complex with HIF-2α (Wu et al., 2014). These results establish HIF-2α as a negative regulator of peroxisome abundance and metabolism.

### 2.3 Structural and functional diversity of HIF-3α

HIF-3α is a member of a diverse protein family. Partial HIF-3α splice variants negatively regulate the hypoxic response. Research indicates that both HIF-3α silencing and Hypoxia-inducible factor prolyl hydroxylase inhibitors (HIF-PHIs) protect cardiomyocytes from hypoxia-reoxygenation injury while restricting expression of classic HIF-1α target genes (Drevytska et al., 2018). Consistent with this study, the truncated HIF-3α4 variant acts as a dominant-negative regulator of HIF-1α and HIF-2α (Maynard et al., 2005). In brief, HIF-3α4 competitively binds HIF-1β via its bHLH-PAS domain, interfering with HIF-1α or HIF-2α dimerization. Lacking a transcription activation domain (TAD), the resulting dimer fails to activate transcription, thereby suppressing the hypoxic response (López-Mejía et al., 2025). Previous studies have demonstrated that HIF-3α2 negatively regulates the hypoxic response, as its overexpression reduces VEGFA expression (Augstein et al., 2011). Inhibitory PAS (Per/Arnt/Sim) domain proteins (IPAS) are another class of HIF-3α variants. Acting as dominant-negative transcription factors for HIF-1α, they suppress angiogenesis while also exerting pro-apoptotic effects (Torii et al., 2011). Additionally, HIF-3α2 is a transcriptional activator involved in erythropoietin production (Tolonen et al., 2020). Other variants are structurally diverse, and their functions remain poorly understood. Table 1 summarizes the splice variants of HIF-1α and HIF-3α.

**TABLE 1:** Summary of HIF splice variants.

HIF variants	Structural features	Species	Main findings	Refs.
HIF-1αTAG,HIF-1α736	Contains a three-base pair TAG insertion between exon 1 and exon 2	human	Significantly elevated in estrogen receptor-negative cancers The mRNA levels of the HIF-1αTAG variant can reflect the degree of breast cancer progression	Dales et al. (2010)
HIF-3α1	Long variant containing LZIP but lacking C-TAD	human	Critically drives CRC progression by promoting EMT, iron accumulation, and metastasis through ZEB2 and TFRC regulation	Xue et al. (2016), Villareal et al. (2024)
HIF-3α2	Long variant starting from exon 1a and containing N-TAD	human	HIF-3α2 is a transcription activator that directly regulates EPO expression Overexpression impairs embryonic growth, developmental timing, and left-right asymmetry Decreases Wnt/β-catenin signaling (independent of HRE-dependent transcription) Decreases hypoxia-mediated expression of VEGFA and Enolase2	Augstein et al. (2011), Tolonen et al. (2020), Tanaka et al. (2009), Zhang et al. (2016), Jaskiewicz et al. (2022)
HIF-3α3	—	—	Predicted to exist but not experimentally confirmed	Pasanen et al. (2010)
HIF-3α4	Short variant similar to mouse IPAS, lacking ODDD, N-TAD, and C-TAD	human	Forms an abortive transcriptional complex with HIF-2α, preventing HIF-2 engagement with HREs Inducible expression impairs angiogenesis, proliferation, and metabolism/oxidation in hypervascular meningiomas	Maynard et al. (2005), Tanaka et al. (2009), Maynard et al. (2007), Heikkilä et al. (2011), Ando et al. (2013), a et al. (2023)
HIF-3α5	—	—	Predicted to exist but not experimentally confirmed	Pasanen et al. (2010)
HIF-3α6	—	—	Predicted to exist, but the sequence contains abnormal repeats and is considered unreliable	Pasanen et al. (2010)
HIF-3α7	Newly identified variant starting from exon 1b and containing a unique exon 15	human	—	Pasanen et al. (2010)
HIF-3α8	Newly identified variant starting from exon 1b and containing LZIP	human	—	Pasanen et al. (2010)
HIF-3α9	Newly identified variant starting from exon 1a and containing LZIP	human	—	Pasanen et al. (2010)
HIF-3α10	Compared to HIF-3α1, it retains intron 1, contains a premature stop codon, and encodes a non-functional 7-amino-acid peptide	human	Predicted to be a regulatory non-coding transcript involved in gene expression control	Pasanen et al. (2010)
IPAS	Contains three unique exons (1a, 4a, and 16). Shares exons 2, 4, and 5 with HIF-3α, along with alternatively spliced variants of exons 3 and 6	mouse	Functions as a dominant-negative transcription factor repressing HIF-1 activity Acts as a dual-function protein mediating transcriptional repression and apoptosis	Torii et al. (2011), Makino et al. (2002), Makino et al. (2007)
NEPAS	Mouse variant encoding a 664-amino-acid polypeptide; differs from HIF-3α only in that exon 1b is replaced by the IPAS exon 1a	mouse	Expressed mainly during embryonic and neonatal stages Dimerizes with HIF-β and suppresses the activity of HIF-1 and HIF-2 Mice with a targeted disruption of the Nepas/HIF-3α locus are viable but exhibit abnormal heart development and lung remodeling	Yamashita et al. (2008)

Abbreviations: bHLH: basic helix-loop-helix; C-TAD: C-terminal transactivation domain; N-TAD: N-terminal transactivation domain; ODDD: oxygen-dependent degradation domain; CRC: colorectal cancer; EMT: epithelial-mesenchymal transition; HRE: hypoxia response element; IPAS: Inhibitory PAS (Per/Arnt/Sim) domain protein; NEPAS: neonatal and embryonic PAS; LZIP: leucine zipper.

### 2.4 Independent oxygen-sensing pathways of PHDs

PHDs continuously hydroxylate HIFs under aerobic conditions, leading to their degradation. In this context, Alex von Kriegsheim et al. demonstrated that p53 is a PHD3 substrate and that PHD3 hydroxylation regulates p53 protein stability by modulating ubiquitination. In brief, they reported that hydroxylation of p53 at Pro359 decreases its association with deubiquitinating enzymes and increases p53 ubiquitination. This modification rapidly reduces p53 protein levels independently of mRNAs expression (Rodriguez et al., 2018). Consequently, they demonstrated that PHD3 functions as an oxygen sensor, regulating p53 to influence cellular behavior through a HIF-independent hypoxic response. Similarly, Liang Xie et al. evaluated the deficiency in PHD2/3’s ability to block mitochondrial uptake and β-oxidation of long-chain fatty acids (LCFAs) in cardiomyocytes. In mice fed a high-fat diet, PHD2/3 deficiency promotes cardiac glucose utilization but exacerbates cardiac dysfunction. PHD2/3 interacts with carnitine palmitoyltransferase 1B (CPT1B), thereby promoting the hydroxylation of CPT1B at P295 to restore LCFA metabolism in PHD2/3-deficient cardiomyocytes (Angelin et al., 2021). Collaboratively, their studies reveal an oxygen-sensitive regulatory axis independent of HIFs.

## 3 HIFs in heart failure: mechanisms and implications

HIFs are central to the critical aspects of HF. However, only a subset of these has been extensively studied to determine the consequences of HIF dysregulation, as described below.

### 3.1 HIF-mediated metabolic remodeling in heart failure

#### 3.1.1 Hierarchical HIFs activation in hypoxia-induced metabolic remodeling

Hypoxic metabolic remodeling and HIF activation are key cardiac adaptations (Su et al., 2021). HIF-1α and HIF-2α differ in their temporal responses to hypoxia: HIF-1α mediates the acute phase (<24 h), while HIF-2α sustains the chronic phase (>24 h) (Koh and Powis, 2012). For example, in cardiac fibroblasts, 4 h of hypoxia activate HIF-1α, but its levels subsequently decline by 24 h. In contrast, HIF-2α remains unresponsive to acute hypoxia, with its nuclear localization unaltered after both 4-h and 24-h exposures (Khalil et al., 2024). As previously mentioned, HIF-1α drives metabolic shifts toward glycolysis, whereas HIF-2α is associated with lipid metabolism. The effects of switches in HIF-1/HIF-2 signaling in chronic hypoxia have not been fully investigated; thus, we need to await further data on their potential implications.

#### 3.1.2 HIF-1α-driven glycolytic shift ETC remodeling in acute hypoxia

**FIGURE 1:**
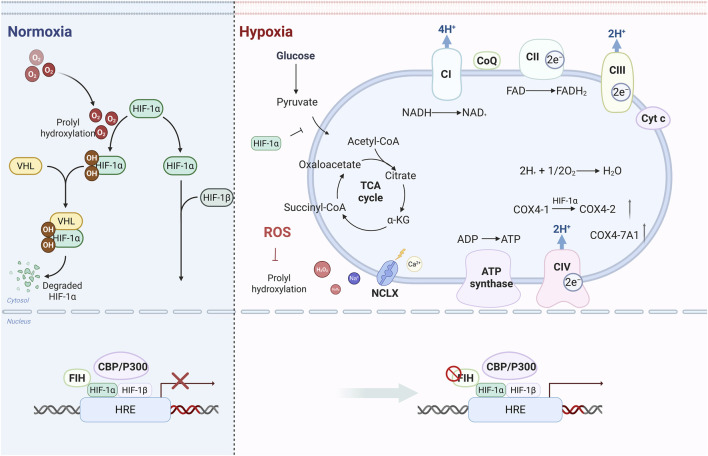
HIF-1α-driven glycolytic shift, ETC remodeling in acute hypoxia. Under normoxia, PHDs hydroxylate HIF-α, targeting it for degradation. Hypoxia inhibits PHDs, stabilizing HIF-α to dimerize with HIF-1β and activate target genes. HIF-1α induces PDK1, which blocks PDH to limit mitochondrial pyruvate entry. It also remodels the ETC via subunit switches (e.g., COX4-2 replacing COX4-1). ROS stabilizes HIF-1α by inhibiting PHDs; hypoxia-induced mitochondrial ROS, mediated by the sodium-calcium exchanger, reinforces this effect.

The heart metabolizes multiple substrates, including fatty acids, glucose, and pyruvate (Ritterhoff and Tian, 2017). Under normal fasting conditions, fatty acid metabolism serves as the primary energy source (Gibb and Hill, 2018). In HF, however, fatty acid utilization is significantly reduced, mediated by PPAR-γ activation, which shifts metabolism from fatty acid oxidation toward lipid storage (Montaigne et al., 2021). This process is driven by HIF-1α, which upregulates PPAR-γ to promote glycolysis (Liu Y. et al., 2022; Krishnan et al., 2009). Specifically, HIF-1α selectively upregulates glycolytic enzyme genes while inhibiting mitochondrial oxidative phosphorylation (Semenza, 2014b; Papandreou et al., 2006). In hypoxia, HIF-1α upregulates pyruvate dehydrogenase kinase 1 (PDK1). This inhibits PDH activity, preventing the conversion of pyruvate to acetyl-CoA and its subsequent mitochondrial entry (Kim et al., 2006). Hypoxia also remodels the mitochondrial electron transport chain (ETC) via HIF-1α-dependent targets to match oxygen consumption with reduced availability. For instance, it prompts the replacement of COX4-1 by COX4-2 in complex IV (Fukuda et al., 2007). HIF-1α markedly upregulates the complex IV subunit COX7a1 in hypoxia, which may facilitate adaptation in the heart (Hwang et al., 2015). Additionally, HIF-1α further induces NDUFA4L2 expression, thereby inhibiting Complex I activity, reducing oxygen consumption, and mitigating ROS accumulation (Tello et al., 2011). These findings identify a mechanism governing HIF-1α-driven metabolic adaptations during acute hypoxia and highlight the therapeutic potential for understanding their broader implications in the progression of HF. Figure 1 shows the role of HIF-1α in acute hypoxia.

#### 3.1.3 Potential beneficial effects of transient to sustained HIF-1α activation in heart failure

Transient-to-sustained HIF-1α activation in HF confers potential protective effects. In a heart-specific HIF-1α transgenic mouse model, HIF-1α expression increases capillary area and enhances glycolysis, thereby boosting cardiac glucose uptake (Hölscher et al., 2012). Reduced mitochondrial pyruvate oxidation and increased lactate efflux are early hallmarks of cardiac hypertrophy and failure. Knockout of the mitochondrial pyruvate carrier (MPC) in adult mouse cardiomyocytes induces myocardial hypertrophy and HF. Conversely, inhibition of the lactate efflux transporter monocarboxylate transporter 4 (MCT4) reverses the hypertrophic phenotype (Cluntun et al., 2021). Consistent with these findings, another study showed that HIF-1α activation upregulates MCT1 and MPC1, thereby delaying HF progression (Xu et al., 2024). These findings suggest that HIF-1α drives beneficial metabolic adaptation.

#### 3.1.4 Dual effects of sustained HIF-1α activation in heart failure progression

Hypoxia activates cellular metabolic adaptation to balance energy demands by accumulating HIF-1α (Su et al., 2021). Indeed, experimental data show that HIF-1α is upregulated throughout the course of HF. In rats with stress-induced HF, early activation and sustained elevation of HIF-1α were observed from 2 to 18 weeks post-surgery. This progression was consistently accompanied by reduced aerobic metabolism and markedly increased glycolysis (Sant’ana et al., 2023). Although adaptive in the short term, sustained HIF-1α activation is highly deleterious.

Several studies have confirmed that sustained glycolytic elevation mediates compensatory cardiac hypertrophy through HIF-1α (Kashihara et al., 2022; Wang M. et al., 2025). Under acute pressure overload, HIF-1α promotes compensatory hypertrophy by enhancing glycolysis and GLUT1 expression (Kashihara et al., 2022). Increased HIF-1α protein levels have been observed in samples from patients with cardiomyopathy. In a cardiac-specific HIF-1α transgenic mouse model, chronic HIF-1α stabilization shifts cardiac metabolism toward glycolysis, increasing glucose uptake. However, under increased mechanical load and aging, this metabolic shift becomes detrimental to cardiac function (Hölscher et al., 2012).

In contrast, conflicting data also exist. For instance, cardiac-specific PHD3 overexpression in mice attenuated HIF-1α accumulation, thereby alleviating cardiac dysfunction and remodeling induced by chronic intermittent hypoxia (Xu et al., 2022). Overall, persistent hypoxia without adequate HIF activation may underlie HF development (Oka et al., 2014). This highlights the dual effects of sustained HIF-1α activation in countering HF.

### 3.2 HIFs regulation of angiogenesis in heart failure

Angiogenesis is defined as the process of forming new blood vessels from preexisting vascular networks (Riley and Smart, 2011). A mismatch between sufficient angiogenesis and excessive myocyte proliferation may represent a pivotal mechanism governing the shift from adaptive hypertrophy to HF (Guo et al., 2015).

#### 3.2.1 HIF-1α-mediated angiogenesis

HIF-1α-mediated angiogenesis influences the prognosis of HF. Elevated expression of HIF-1α and VEGFA enhances cardiac function by promoting cardiac angiogenesis (Wu X. et al., 2021). VEGF augments vascular permeability, enabling plasma proteins to extravasate and establish a provisional scaffold for endothelial cell migration (Eliceiri et al., 1999). Endothelial cells then migrate distally, aggregate into solid cord-like formations, and ultimately undergo lumenization. Upon vessel maturation, pericytes and smooth muscle cells envelop the nascent capillaries to reinforce vascular stability. Several studies have demonstrated that sustained HIF-1α activation may confer protective effects in HF by promoting angiogenesis and mitigating pathological remodeling. In animal models, overexpression of HIF-1α in various cardiac cell types has been shown to enhance angiogenesis and improve overall cardiac function in mice with HF (Tang et al., 2021; Li et al., 2021). For instance, in post-myocardial infarction (MI) settings, enhanced endogenous angiogenesis driven by HIF-1α reduces scar formation and attenuates adverse left ventricular remodeling (Wu X. et al., 2021). Consistently, in a pressure-overload HF model induced by transverse aortic constriction (TAC), combined chronic alveolar hypoxia activates HIF-1α-mediated pathways, leading to increased angiogenesis, reduced cardiac hypertrophy, and suppressed expression of genes linked to maladaptive remodeling, ultimately counteracting the progression to HF (Froese et al., 2024). At the cellular level, these benefits appear to be linked to HIF-1α′s regulation of angiogenic factors. Prolonged hypoxia in cardiomyocytes can paradoxically suppress angiogenesis by activating p53, which degrades HIF-1α protein (He et al., 2024; Sano et al., 2007). In this context, supplementation with HIF-1α-regulated growth factors has been shown to prevent the transition from compensatory hypertrophy to maladaptive ventricular dilatation and HF (Guo et al., 2015). While clinical evidence remains limited, these findings collectively support a potential cardioprotective function for cardiomyocyte-specific HIF-1α activation in regulating angiogenesis and halting HF progression, warranting further translational research.

#### 3.2.2 HIF-2α-mediated angiogenesis

HIF-2α suppresses excessive angiogenesis and promotes vascular maturation (Bakleh and Al Haj Zen, 2025). Endothelial HIF-2α knockout leads to increased angiogenesis, but the resulting arteries are functionally impaired (Skuli et al., 2012). Recent studies have reported that HIF-2α inhibits microvascular proliferation. Systemic HIF-2α inhibition under chronic hypoxia induces excessive capillary proliferation, resulting in cardiac hypertrophy and failure during chronic hypoxia. This hypertrophic effect arises not from increased cardiomyocyte size but from abnormal remodeling (lumen dilation) of cardiac capillaries, which disrupts the cardiac microenvironment. Notably, cardiac anomalies associated with HIF-2α deficiency resolve upon reoxygenation. Thus, during chronic hypoxia, HIF-2α attenuates pathological microvascular proliferation and remodeling to preserve vascular integrity, a process modulated by oxygen levels and reversible (Albendea-Gomez et al., 2025). Persistent HIF-2α inhibitory signaling may explain the dysregulated proliferation and vascular sparseness observed in myocardial capillary networks during HF. These findings align with autopsy observations in Heart Failure with Preserved Ejection Fraction (HFpEF) patients, showing widespread cardiac hypertrophy, coronary artery disease, microvascular sparseness, and myocardial fibrosis (Mohammed et al., 2015). Selectively modulating HIF-2α activity in response to the duration of hypoxia may serve as a therapeutic strategy for HF.

### 3.3 HIFs regulation of inflammatory responses in heart failure

In HF, the immune system initially mounts an acute, localized inflammatory response aimed at tissue repair. However, dysregulation over time can transform this response into a chronic, systemic inflammatory state. Consequently, its effects shift from protective to destructive and extend from the initial infarct zone to the entire myocardium (Chen et al., 2024; Wang et al., 2019). The interplay between inflammation and HIFs is a pivotal factor in the pathophysiology of HF. HIFs enable immune cells to adapt to hypoxic conditions commonly found in inflamed tissues (MCGettrick and O’Neill, 2020).

#### 3.3.1 HIF-1α and HIF-2α in regulating inflammatory responses in heart failure

Macrophages are central to the initial inflammatory response following cardiovascular injury, where they contribute to both tissue damage and repair processes in HF (Chen et al., 2024; Lunderberg et al., 2025). In this context, HIF isoforms play distinct roles in macrophage polarization and function. HIF-1α predominantly drives pro-inflammatory M1 macrophages by upregulating inducible nitric oxide synthase (iNOS) expression, while HIF-2α promotes anti-inflammatory M2 macrophages through enhanced arginase-1 activity. These pathways compete for the shared substrate L-arginine. HIF-1α antagonizes M2/Arg1 responses while HIF-2α counters M1/iNOS activity, thereby tuning the inflammatory balance (Takeda et al., 2010). Furthermore, HIF-mediated metabolic remodeling in macrophages influences HF progression. Modulating this remodeling has been shown to protect against MI-induced cardiac remodeling (Lin et al., 2024). For example, in post-MI remodeling, Ly6C^hi^ macrophages inhibit cardiac fibroblast activation *via* HIF-1α-dependent suppression of the TGF-β/SMAD signaling pathway, thereby reducing fibrosis and adverse remodeling (ABE et al., 2019). Additionally, the macrophage-specific protein Allogeneic Inflammatory Factor 1 enhances HIF-1α activation by promoting actin remodeling, which shifts metabolism toward glycolysis. This fuels sustained inflammation, exacerbating left ventricular remodeling and HF progression (Deberge et al., 2025). These findings underscore the macrophage-centric roles of HIF-1α and HIF-2α in HF inflammation.

Cytotoxic CD8^+^ T cells modulate inflammatory responses. These cells are recruited to ischemic heart tissue, where they activate and release Granzyme B, inducing cardiomyocyte apoptosis, adverse ventricular remodeling, and myocardial dysfunction. Depleting CD8^+^ T cells reduces apoptosis, dampens inflammation, limits injury, and improves heart function (Santos-Zas et al., 2021). The interaction between CD8^+^ T cells and HIFs is a crucial factor in cardiac remodeling (Doedens et al., 2013; Dvorakova et al., 2024). For instance, Yingjie Chen et al. showed that HIF-1α stabilization enhances CD8^+^ T cell activation and cytokine production, thereby exacerbating cardiopulmonary inflammation, fibrosis, and dysfunction (Pan et al., 2025). While PHD2 deletion in CD8^+^ T cells has no effects on heart function or effector molecules under baseline conditions, it exacerbates remodeling after pressure overload by promoting activation and cytokine release through a HIF-1α-dependent pathway (Santos-Zas et al., 2021). Their findings indicate that HIF-1α-mediated regulation of CD8^+^ T cells amplifies inflammation and remodeling in response to cardiac stress.

**FIGURE 2:**
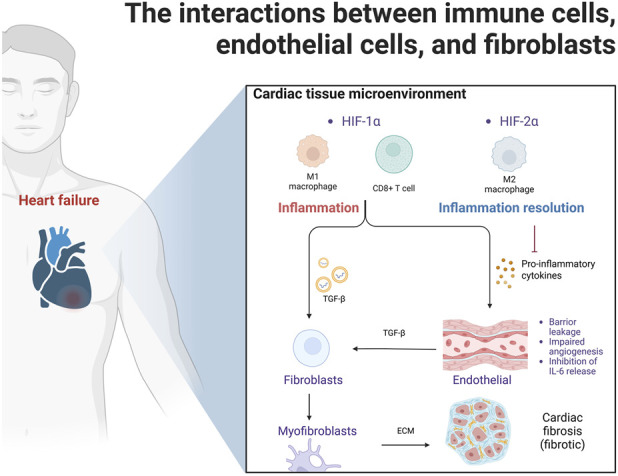
The interactions among immune cells, endothelial cells, and fibroblasts. Initial injury activates HIF-1α-driven M1 macrophages and CD8^+^ T cells, which attack endothelial cells, causing dysfunction. Damaged endothelium and pro-inflammatory signals activate TGF-β/Smad signaling, transforming fibroblasts into myofibroblasts that secrete excess extracellular matrix, leading to fibrosis, adverse remodeling, and heart failure progression. In contrast, HIF-2α exerts anti-inflammatory effects by promoting M2 macrophages (via Arg-1) and directly inhibiting inflammatory factor expression in endothelial cells.

As previously mentioned, stromal cells, such as endothelial cells and fibroblasts, contribute to HIF-mediated inflammation in HF, driving processes such as tissue damage, repair, and angiogenesis. HIF-1α activates the TGF-β/Smad signaling pathway in cardiac fibroblasts, promoting fibrosis and cardiac remodeling (Feng et al., 2021). In contrast, anti-inflammatory HIF-2α in endothelial cells helps counterbalance these effects, potentially preserving endothelial integrity (Ullah et al., 2025). In this context, immune cells interact dynamically with endothelial cells and fibroblasts via HIF signaling, serving as a key driver of pathological remodeling. Among the available evidence, the strongest findings stem from *in vivo* models (Li et al., 2021; Ullah et al., 2025), which suggest that the key regulatory focus of HIFs may lie not in cardiomyocytes but rather in endothelial cells. Figure 2 shows the interactions among immune cells, endothelial cells, and fibroblasts.

#### 3.3.2 ARNT in regulating inflammatory responses in heart failure

ARNT, a core member of the bHLH-PAS protein family lacking intrinsic oxygen sensitivity, plays a pivotal role in modulating inflammatory responses in HF by interacting with NF-κB pathways and HIF-2α. ARNT forms a complex with the RelB subunit of the NF-κB family (ARNT/RelB), which antagonizes the pro-inflammatory RelA/p50 complex, thereby suppressing the production of inflammatory mediators such as IL-6 (Wright and Duckett, 2009). Recent studies further demonstrate that ARNT can directly bind to the IL-6 gene promoter to inhibit its transcription, highlighting its direct anti-inflammatory action. Evidence from cellular models underscores ARNT’s protective effects in cardiac endothelium. In human cardiac microvascular endothelial cells deficient in HIF-2α, there is impaired barrier integrity, reduced expression of tight junction proteins, increased cell death, and elevated IL-6 levels. Notably, overexpression of ARNT restores and protects cardiac microvascular barrier integrity. However, this cardioprotective effect is abolished following HIF-2α deletion, even when ARNT protein levels remain unchanged (Ullah et al., 2025). These findings suggest that under hypoxic conditions prevalent in HF, ARNT’s function shifts from a constitutively expressed co-activator to an HIF-2α-dependent suppressor of inflammation. Directly targeting the enhancement of ARNT’s inhibitory activity on the IL-6 promoter, rather than modulating the unstable HIF-2α protein, may offer a novel approach for developing cardioprotective drugs.

### 3.4 Circadian influences on HIFs and heart failure

Circadian rhythms exert profound influences on cardiovascular function and disease progression (Sole and Martino, 2023; Zhang and Jain, 2021). Clinical studies have shown that diurnal patterns in the severity of cardiac injury following MI (Reiter et al., 2012; Thosar et al., 2018). However, the molecular mechanisms underlying these diurnal variations remain unclear in HF. Emerging evidence suggests that HIFs play a pivotal role in mediating these diurnal variations, potentially through interconnected pathways involving metabolic adaptation and circadian clock components. In HF patients, transcript levels of Apaf-1-interacting protein (APIP) and adenosine receptor A2B (ADORA2B) are significantly elevated compared to controls, and their mRNAs show a strong correlation (Lim et al., 2019). APIP mitigates ischemic cardiac injury by binding to and stabilizing ADORA2B expression (Lim et al., 2019), which, in turn, promotes adaptive cardiac responses. Genetic models further reveal that ADORA2B signaling attenuates MI by enhancing metabolic efficiency, enabling the heart to utilize carbohydrates more effectively during stress (Eltzschig et al., 2013). Furthermore, ADORA2B stabilizes the circadian rhythm protein Period 2 (Per2) under myocardial ischemia, while Per2(−/−) mice display larger infarct sizes, impaired HIF-1α stabilization, and reduced glycolytic capacity (Eckle et al., 2012). Intense light exposure amplifies Per2 oscillations and confers cardioprotection under normoxic conditions, recapitulating hypoxia- and HIF-1α-driven metabolic adaptations (Oyama et al., 2019). Consistent with these findings, the core circadian transcription factor BMAL1 regulates diurnal myocardial injury by forming a transcriptionally active heterodimer with HIF-2α. This interaction drives circadian-dependent gene expression, with BMAL1 target genes such as Per2, Per3, and amphiregulin showing significant upregulation at Zeitgeber time 8. These findings identify a mechanism governing circadian variations of myocardial injury and highlight the therapeutic potential of clock-based pharmacological interventions for treating ischaemic heart disease, such as HF.

### 3.5 Oxygen-sensing hierarchy in adaptive hypoxic responses for heart failure

Inhibiting PHDs and FIH may offer a therapeutic strategy for ischemic HF by modulating the oxygen-sensing hierarchy. As previously mentioned, PHDs exist as distinct isoforms, with expression patterns varying significantly across tissues. The heart primarily expresses PHD2 and PHD3 (Willam et al., 2006). FIH is another oxygen sensor that hydroxylates the C-terminal transactivation domain of HIF proteins, blocking their binding to the coactivator p300/CBP (Wu Y. et al., 2021). With a lower Km for oxygen than PHDs, FIH requires deeper hypoxia for inhibition, thus remaining active under mild hypoxia to suppress gene transcription despite HIFs’ accumulation. In HF progression, this hierarchy can yield beneficial outcomes under controlled conditions. For instance, recent research has linked enhanced recruitment of bone marrow cells into ischemic myocardium in PHD2/FIH double-knockout mice to elevated expression of angiogenic factors, increased neovascularization, and improved cardiac function (Huang et al., 2011). Similarly, Ang-1 gene therapy in leptin receptor gene-deficient mice suppresses PHD2, boosts Akt and eNOS, and markedly increases capillary/arterial density while reducing hypertrophy and fibrosis at 14 days post-ischemia (Chen and Stinnett, 2008). Thymosin beta-4 transfection in BMMSCs further promotes HIF-1α stability via the AKT pathway and inhibits its degradation by PHD/FIH, thereby accelerating ischemic recovery (Tang et al., 2021). However, this hierarchical hypoxia sensing cannot prevent sustained HIF activation during severe hypoxia, which can be detrimental. For example, PHD2 expression is significantly reduced in cardiac tissue and cardiovascular endothelial cells from patients with cardiomyopathy (DAI et al., 2021), potentially leading to pathological overactivation. Therefore, targeting this oxygen-sensing layer in HF must balance adaptive HIF outputs with the risk of pathological overactivation. Supplementary Figure S1 provides an overview of this regulatory network of HIF signaling in Heart Failure.

## 4 Potential drugs targeting the HIF pathway for treating heart failure

An imbalance in the interaction between HIF-1α and HIF-2α may contribute to chronic HF, characterized by HIF-1α activation and HIF-2α inhibition (PACKER, 2020). Given this dysregulation, numerous pharmacological agents have been identified to modulate HIF signaling, with several demonstrating promising potential for HF treatment.

### 4.1 Hypoxia-inducible factor prolyl hydroxylase inhibitors

HIF-PHIs are a novel therapeutic class that pharmacologically inhibit prolyl hydroxylase domains, thereby stabilizing HIFs. This stabilization promotes erythropoiesis by enhancing red blood cell production. Primarily developed for treating anemia in chronic kidney disease (Li Z. et al., 2025), HIF-PHIs pharmacologically stabilize HIFs. Clinical and animal studies suggest they also influence numerous non-anemia-related genes, potentially leading to complications such as vascular calcification, thrombogenesis, and HF exacerbation (Nakanishi and Kuragano, 2024). Given the potential risks of HF, preliminary evidence from a single-center retrospective analysis suggests additional benefits beyond anemia treatment, including greater reductions in BNP levels compared with oral iron supplementation (YOSHITAKE et al., 2025). However, existing studies are limited by small sample sizes and single-center designs, underscoring the urgent need for research in this field.

### 4.2 Metformin

Metformin is an antidiabetic drug with well-established cardioprotective effects. Although the effects of metformin on HIF-1α signalling have not been investigated directly, the protective effect of metformin in HF may be related to sustained inhibition of HIF-1α. In studies of patients with HF and diabetes, metformin use was associated with significant improvements in clinical outcomes (Khan et al., 2022). *In vivo* studies have demonstrated that metformin reduces cardiac hypertrophy in high-fat diet-fed rats via the HIF-1α/PPAR-γ signaling pathway. Metformin reduced cardiomyocyte volume, and administration of agonists for this pathway markedly abolished the protective effect of metformin on cardiac hypertrophy (Liu Y. et al., 2022). Consequently, patients with comorbid HF and type 2 diabetes, particularly those exhibiting cardiac hypertrophy or at high risk for adverse cardiovascular events, may benefit from treatment with this drug.

### 4.3 Astragaloside IV

Astragaloside IV, the primary active component of the Chinese herb Astragalus membranaceus, exhibits significant cardioprotective effects in HF. AST-IV treatment improved survival rates and cardiac function in HF rats while promoting angiogenesis (Sui et al., 2019). The astragaloside IV derivative HHQ16 improves MI-induced myocardial hypertrophy and HF by degrading lncRNA4012/9456 (Wan et al., 2023). These cardioprotective effects of Astragaloside IV in HF are closely linked to the modulation of HIF signaling and energy metabolism pathways. It has been reported that astragaloside IV alleviates HF by activating PPARα and shifting metabolism from glycolysis to fatty acid β-oxidation (Dong et al., 2017). Another study indicates that astragaloside IV modulates energy metabolism and restores mitochondrial function by regulating the HIF-1α/PPARγ pathway in a heart failure-like model, thereby protecting the heart (Li Q. et al., 2025).

Furthermore, Astragaloside IV has been shown to influence HIF-2α signaling in HF contexts. In the HF rat model, AST-IV significantly increased HIF-2α and Rho/ROCK pathway protein expression, thereby enhancing cell proliferation and reducing apoptosis and ROS levels. The protective effects were partially reversed by ROCK inhibitors or HIF-2α knockdown (Li G. et al., 2025). Collectively, these studies demonstrate that AST-IV may regulate both HIF-1α and HIF-2α in HF. Therefore, Astragaloside IV could be suitable for patients with ischemic or metabolic heart failure, targeting endpoints such as improved cardiac function, reduced hypertrophy, and enhanced survival.

### 4.4 Silybin

Silybin is a flavolignan compound extracted from plants of the Asteraceae family. Previous studies have demonstrated that silybin possesses hepatoprotective and metabolic regulatory effects (Colturato et al., 2012). However, its pharmacological actions are not limited to these. Multiple studies indicate that silibinin reduces HIF-1α levels in cancer cells and inhibits angiogenesis (Gu et al., 2007; Singh et al., 2008; Sellam et al., 2020). Furthermore, silybin treatment improves cardiac function and limits infarct size, thereby inhibiting fibroblast remodeling in mice with myocardial ischemia-reperfusion injury (Chen et al., 2020). Therefore, it can be hypothesized that HIFs are among its therapeutic targets in heart failure. A recent study indicates that silybin improves post-myocardial infarction heart failure (post-MI HF) by regulating energy metabolism. In animal experiments, silybin treatment significantly enhanced cardiac function parameters in mice with post-MI HF, reduced left ventricular dilatation, and decreased myocardial fibrosis. Both silybin and HIF-1 inhibitors downregulated HIF-1α, GLUT1, and LDHA mRNAs and protein expression in post-MI HF, elevated ATP levels, and reduced lactate production and ROS accumulation. This confirms that silybin’s protective effect against post-MI HF depends on HIF-1α (Wang M. et al., 2025). These findings support the role of silybin in enhancing the therapeutic response to and improving outcomes in post-MI HF, although further research is needed.

### 4.5 Allicin

Allicin is a sulfur-containing organic compound primarily derived from garlic. Research highlights its roles in modulating cholesterol metabolism and attenuating oxidative stress (Liang et al., 2025; Chen et al., 2025; Huang et al., 2026). An experimental study assessed whether allicin attenuates vascular remodeling and RV hypertrophy in pulmonary arterial hypertension (PAH). This protective effect may be mediated through NO-dependent vasodilation, modulation of the RAS, and amelioration of OS. These effects could be associated with HIF-1α modulation and improved lung oxygenation (Sánchez-Gloria et al., 2023). Although human studies are needed, the data suggest that, alone or in combination therapy, allicin may be an alternative in treating PAH and HF.

### 4.6 Raspberry polyphenols

Raspberries are rich in polyphenols, which may favorably impact enzymes involved in redox homeostasis and target inflammatory signaling. *In vivo* studies showed that a 7-week raspberry diet (initiated 3 weeks before HF induction) significantly reduced oxidative stress, apoptosis, and cardiac remodeling. *In vitro* studies showed that a raspberry polyphenol extract intervention in a CoCl_2_-induced chemical hypoxia model of human cardiomyocytes alleviated oxidative stress and apoptosis despite pronounced HIF-1α expression (Najjar et al., 2024). These findings indicate that the primary cardioprotective mechanisms of raspberry polyphenols are either independent of the HIF-1α pathway or act downstream of it. This positioning enables them to counteract pathological processes driven by excessive HIF-1α activation or by alternative pathways. Thus, a raspberry-based dietary intervention may represent a promising alternative or adjunctive strategy for the management of heart failure, although further validation in diverse HF models is warranted.

### 4.7 Draconis sanguis

Draconis sanguis (DS), which promotes blood circulation and dissolves stasis, has been investigated in the treatment of ischemic cardiovascular diseases. It has been demonstrated that DS can inhibit ox-LDL-induced oxidative stress in vascular endothelial cells and reduce the severity of atherosclerotic lesions in ApoE(−/−) mice (Zhao et al., 2024). Its extract significantly inhibits NO-induced monocyte-endothelial cell adhesion (Wang et al., 2026). Experimental studies show that DS exerts cardioprotective effects in ischemic conditions, in part by modulating HIF signaling pathways. Chun Li et al. found that DS improves cardiac function by optimizing myocardial energy metabolism after promoting angiogenesis *via* regulation of the HIF-1α/VEGF signaling pathway (Li J. et al., 2025). In a rat model of heart failure induced by coronary artery ligation, treatment with DS increased the 28-day survival rate by 10%, elevated the left ventricular ejection fraction by 20%, and significantly reduced myocardial tissue damage. The active compounds in DS upregulate HIF-1α expression, thereby promoting angiogenesis and increasing local oxygen delivery. This shifts myocardial energy metabolism towards the production and protection of vascular endothelial cells. Notably, DS-d (4-methylpinosylvin) demonstrated the highest binding affinity to the HIF-1α protein, significantly upregulating HIF-1α mRNAs expression and enhancing cell viability in HUVECs under hypoxic conditions (Li J. et al., 2025). DS-d (4-methylpinosylvin) may serve as the primary active substance through which Draconis Sanguis ameliorates ischemic heart failure via the HIF-1α/VEGF signaling pathway, thus highlighting its clinical translation potential.

### 4.8 Traditional Chinese Medicine formulas

Traditional Chinese Medicine (TCM) formulas can improve HF through HIFs-related pathways, though their regulatory effects are highly context-dependent. Currently, the evidence is primarily preclinical, requiring rigorous validation for clinical translation regarding efficacy and safety.

TCM formulas exert bidirectional, multi-target regulatory effects on HIFs. For instance, Shenfu injection suppresses glycolysis *via* the HIF-1α pathway, thereby alleviating myocardial fibrosis in HF mice/rats (Li X. et al., 2025; Ouyang et al., 2025). Similarly, An Pan et al. found that the Xinkeshu formula reduced HIF-1α accumulation and nuclear translocation *via* the AMPK/mTOR cascade, based on *in vivo* and *in vitro* data, thereby inhibiting glycolysis and mitochondrial oxidation while suppressing pathological cardiac hypertrophy (Zhao et al., 2025). Notably, certain TCM formulas promote angiogenesis and regulate myocardial energy metabolism by activating HIF-1α. For instance, the active components of Linggui Zhugan Decoction modulate HIF-1α and HO-1, upregulating autophagy markers while downregulating p62, thereby enhancing compensatory autophagy and mitigating myocardial injury (Ren et al., 2024). Similarly, Shen’ge Formula improved cardiac function and alleviated myocardial hypertrophy in stress-induced heart failure rats via Akt activation, HIF-1α upregulation, and inhibition of p53 nuclear translocation (Qiu et al., 2024). Yong Wang et al. found that Danqi Pill protects against post-MI HF by activating the HIF-1α/PGC-1α pathway. This activation upregulates GLUT4 and PKM2, promotes mitochondrial biogenesis via NRF1 and TFAM, and elevates ATP levels, with gene knockdown confirming HIF-1α as the key upstream target (Zhang et al., 2020). Research on Qiliqiangxin has also revealed that it promotes fatty acid uptake/utilization via CD36 and PGC-1α upregulation, while boosting microvascular density and blood supply through the HIF-1α/VEGF pathway (Wang et al., 2020). Collectively, these findings highlight the central role of HIF-1α as a common target through which diverse TCM formulas exert cardioprotection.

On the other hand, some TCM formulas exhibit opposite effects on HIF-1α in the border and remote areas of the heart. Qishen Granules promoted HIF-1α expression and its entry into the nucleus under high levels of hypoxia, thereby enhancing compensatory glucose metabolism, while reducing nuclear accumulation of HIF-1α under relatively low levels of hypoxia, promoting increased lipid metabolism, and improving cardiac function in HF rats (Sun et al., 2025). In addition, multiple components in TCM formulas demonstrate direct or indirect regulatory effects on the HIF-1α pathway. For example, Qishen Yiqi Dripping Pills are used clinically to treat various myocardial ischemic diseases. Their therapeutic efficacy is achieved through multiple targets and pathways, with the HIF-1α signaling pathway serving as a critical one. Ginsenoside Rg1, an active ingredient in Qishen Yiqi Dripping Pills, significantly downregulated the expression of HIF-1α protein and upregulated the expression of VEGFA. Furthermore, Salvianic acid A significantly downregulated the protein expression of the upstream phosphatidylinositol-4,5-bisphosphate 3-kinase catalytic subunit alpha and the downstream HIF-1α in the Akt1 pathway (Gong et al., 2021). Table 2 comprehensively lists natural compounds targeting HIFs for the treatment of HF based on a broader literature review (including studies on myocardial infarction with similar pathological models and network pharmacology predictions).

**TABLE 2:** Natural compounds targeting the HIFs for treating HF.

Compounds	HIF signaling	Type of study	Main findings	Refs
Astragaloside IV	HIF-2α/Rho/ROCK	Rats (HF)	AST-IV increased HIF-2α and Rho/ROCK proteins, enhancing proliferation and reducing apoptosis and ROS	Li et al. (2025c)
Silybin	HIF-1α/PFKFB3/GLUT1	Mice (LAD ligation), HL-1 cardiomyocytes (OGD)	Silybin improves cardiac function in post-MI HF via HIF-1α-mediated glycolysis	Wang et al. (2025b)
Allicin	HIF-1α/VEGF	Rats(PAH)	Allicin attenuates PAH-induced vascular remodeling and RV hypertrophy via HIF-1α/VEGF inhibition	Sánchez-gloria et al. (2023)
Raspberry polyphenols	—	Sprague Dawley rats (LAD ligation), human cardiomyocytes(CoCl2)	RBPE attenuates CoCl_2_-induced oxidative stress and apoptosis in cardiomyocytes despite pronounced HIF-1α expression	Najjar et al. (2024)
Draconis Sanguis	HIF-1α/VEGF	Rats (LAD ligation)	Draconis Sanguis ameliorates myocardial ischemia by upregulating HIF-1α to promote angiogenesis, enhance oxygen supply, and optimize energy metabolism	Li et al. (2025d)
Apigenin	HIF-1α/PPARα/CPT-1	Rats (Hypertension-induced cardiac hypertrophy)	Apigenin directly suppresses HIF-1α expression, reducing collagen production and cardiac fibrosis	Feng et al. (2021)
Terminalia arjuna	—	—	Terminalia arjuna confers cardioprotection via multiple pathways, including HIF signaling	Asghar et al. (2023)
Shenfu injection	HIF-1α/PFKFB3, PI3K-AKT/HIF-1α	Rats (ISO), Mice (TAC)	Downregulation of HIF-1α inhibits myocardial fibrosis	Li et al. (2025e), Ouyang et al. (2025)
Xinkeshu formula	AMPK/mTOR/HIF-1α	Mice (TAC)	Xinkeshu formula inhibits pathological cardiac hypertrophy by modulating metabolic remodeling via the AMPK/mTOR cascade	Zhao et al. (2025)
Danqi Pill	HIF-1α/PGC-1α	Rats (LAD ligation), H9c2 cells (OGD/R)	Danqi Pill drives glycolytic and mitochondrial metabolism via HIF-1α/PGC-1α to improve post-AMI HF	Zhang et al. (2020)
LGZGD	HIF-1α/HO-1	Mice (LAD ligation)	LGZGD enhances autophagy and cardiac function in post-MI CHF by upregulating HIF-1α/HO-1 signaling	Ren et al. (2024)
Shen’ge Formula	p-Akt/Akt/HIF-1α	Rats (AAC)	Shen’ge Formula activates Akt/HIF-1α while inhibiting p53 to combat pressure overload-induced HF	Qiu et al. (2024)
Qiliqiangxin	HIF-1α/VEGF	Rats (LAD ligation)	Qiliqiangxin improves cardiac function via HIF-1α-dependent and -independent regulation of energy metabolism	Wang et al. (2020)
QSG	—	Rats (LAD ligation)/H9c2 cells (hypoxia-induced)	Context-dependent regulation of HIF-1α nuclear translocation	Sun et al. (2025)
QSYQ	HIF-1α/VEGF	H9c2(OGD)	QSYQ active ingredients synergistically protect cardiomyocytes by inhibiting HIF-1 signaling	Gong et al. (2021)
HX	—	Rats (LAD ligation)	HX protects against MI through multitarget regulation, including HIF-1α signaling	Lin et al. (2022)
NXK	HIF-1α/PDK1	Mice (LAD ligation), RAW264.7 cells(LPS)	NXK modulates macrophage metabolism and inflammation via the HIF-1α/PDK1 axis	Lin et al. (2024)
Mongolian medicine Nutmeg-5	—	Kunming mice (LAD ligation)	Nutmeg-5 attenuates post-MI cardiac remodeling by inhibiting HIF-1α to preserve mitochondrial function	Liu et al. (2022b)
TLP	—	—	TLP exerts therapeutic effects in HFpEF via multi-target regulation involving the HIF-1α pathway	Chi et al. (2024)

Abbreviations: H/R: hypoxia-reoxygenation; OGD: oxygen-glucose deprivation; OGD/R: oxygen-glucose deprivation/reoxygenation; Dox-HF: doxorubicin-induced heart failure; LAD ligation: left anterior descending coronary artery ligation; ISO: isoproterenol-induced cardiac injury; TAC: transverse aortic constriction; AAC: ascending aortic constriction; RBPE: raspberry polyphenol extract; LGZGD: linggui zhugan decoction; FFA: free fatty acid; QSG: qishen granules; QSYQ: qishen yiqi dripping pills; HX: huoxue wentong formula; NXK: nuanxinkang; TLP: tingli pill.

The above studies reveal the multifaceted mechanisms by which TCM formulas modulate HF via the HIF pathway. However, large randomized clinical trials are lacking, and thus, prescribing decisions cannot be made. To establish Chinese medicine compound formulas as viable interventions for heart failure, future research should focus on several key directions. Multicenter, large-sample, randomized, double-masked, placebo-controlled clinical trials should be conducted to evaluate their effects on endpoints such as rehospitalization rates, cardiovascular mortality, and quality of life. Clinical studies should also integrate biobanks to monitor relevant biomarkers, thereby dynamically validating preclinical mechanisms. Furthermore, exploring combination therapies with standard HF medications is essential to assess potential synergistic effects, reduced drug resistance, and fewer adverse reactions, offering new options for comprehensive disease management. In summary, while Chinese medicine compounds show therapeutic promise through HIFs-related pathways, their broader clinical application requires more rigorous evidence.

## 5 Conclusion

This review primarily focuses on the role of HIF in the pathophysiology of HF. Whether sustained HIF-1α upregulation benefits or harms the heart in HF remains controversial. Short-term activation is cardioprotective via angiogenesis and metabolic remodeling, whereas chronic activation may drive pathological hypertrophy and metabolic dysregulation. Hierarchical hypoxia is a common feature of malignant tumor microenvironments. Under intensified hypoxia, it drives the sequential activation of HIF-induced glycolysis, immunosuppression, angiogenesis, and necrotic apoptosis (Wang P. et al., 2025). However, this remains unexplored in HF. A graded HIF-1α regulatory mechanism may exist in response to varying hypoxia levels, suggesting an optimal therapeutic window. Nevertheless, current HIF research shows no consistent pattern. This is likely due to diverse HF models and varying hypoxic conditions. Most studies ignore hypoxia gradation and individual tolerance to hypoxia. In summary, HIF activation is tissue-specific, and its role in HF progression depends on disease stage, duration, and cellular context.

## Supplementary material

SUPPLEMENTARY FIGURE S1 The regulatory network of HIF signaling in Heart Failure. Pathological stimuli, such as ischemia-hypoxia and pressure overload, activate the HIF system. The activated HIF isoforms synergistically modulate downstream pathophysiological processes, encompassing metabolic remodeling, angiogenesis, inflammatory responses, and other processes potentially indirectly influenced by the HIF signaling pathway. Meanwhile, this hierarchical regulatory network may exhibit mutually antagonistic effects, which can be beneficial or detrimental to HF progression.

## References

[B1] A R. Kunimura N. Tominaga S. Hirata E. Nishioka S. Uesugi M. (2023). A recombinant adenovirus vector containing the synNotch receptor gene for the treatment of triple-negative breast cancer. Front. Oncol. 13, 1147668. 10.3389/fonc.2023.1147668 37064130 PMC10090503

[B2] Abe H. Takeda N. Isagawa T. Semba H. Nishimura S. Morioka M. S. (2019). Macrophage hypoxia signaling regulates cardiac fibrosis via Oncostatin M. Nat. Commun. 10 (1), 2824. 10.1038/s41467-019-10859-w 31249305 PMC6597788

[B3] Albendea-Gomez T. Mendoza-Tamajon S. Castro-Mecinas R. Escobar B. Ferreira Rocha S. Urra-Balduz S. (2025). Vascular HIF2 signaling prevents cardiomegaly, alveolar congestion, and capillary remodeling during chronic hypoxia. Arterioscler. Thromb. Vasc. Biol. 45 (3), e78–e98. 10.1161/ATVBAHA.124.321780 39846162

[B4] Ando H. Natsume A. Iwami K. Ohka F. Kuchimaru T. Kizaka-Kondoh S. (2013). A hypoxia-inducible factor (HIF)-3α splicing variant, HIF-3α4 impairs angiogenesis in hypervascular malignant meningiomas with epigenetically silenced HIF-3α4. Biochem. Biophys. Res. Commun. 433 (1), 139–144. 10.1016/j.bbrc.2013.02.044 23485455

[B5] Andreadou I. Ghigo A. Nikolaou P. E. Swirski F. K. Thackeray J. T. Heusch G. (2025). Immunometabolism in heart failure. Nat. Rev. Cardiol. 22 (10), 751–772. 10.1038/s41569-025-01165-8 40544171

[B6] Angelini A. Saha P. K. Jain A. Jung S. Y. Mynatt R. L. Pi X. (2021). PHDs/CPT1B/VDAC1 axis regulates long-chain fatty acid oxidation in cardiomyocytes. Cell. Rep. 37 (1), 109767. 10.1016/j.celrep.2021.109767 34610308 PMC8658754

[B7] Asghar A. Qasim M. Noor F. Ashfaq U. A. Tahir Ul Qamar M. Masoud M. S. (2023). Systematic elucidation of the multi-target pharmacological mechanism of Terminalia arjuna against congestive cardiac failure via network pharmacology and molecular modelling approaches. Nat. Prod. Res. 37 (22), 3733–3740. 10.1080/14786419.2023.2252565 37665010

[B8] Augstein A. Poitz D. M. Braun-Dullaeus R. C. Strasser R. H. Schmeisser A. (2011). Cell-specific and hypoxia-dependent regulation of human HIF-3α: inhibition of the expression of HIF target genes in vascular cells. Cell. Mol. Life Sci. 68 (15), 2627–2642. 10.1007/s00018-010-0575-4 21069422 PMC11115058

[B9] Bakleh M. Z. Al Haj Zen A. (2025). The distinct role of HIF-1α and HIF-2α in hypoxia and angiogenesis. Cells 14 (9), 673. 10.3390/cells14090673 40358197 PMC12071368

[B10] Butt J. H. Jhund P. S. Docherty K. F. Claggett B. L. Vaduganathan M. Bachus E. (2024). Dapagliflozin and timing of prior heart failure hospitalization: a patient-level meta-analysis of DAPA-HF and DELIVER. JACC Heart Fail 12 (9), 1586–1599. 10.1016/j.jchf.2024.01.018 38573262

[B11] Chen J. X. Stinnett A. (2008). Ang-1 gene therapy inhibits hypoxia-inducible factor-1alpha (HIF-1alpha)-prolyl-4-hydroxylase-2, stabilizes HIF-1alpha expression, and normalizes immature vasculature in db/db mice. Diabetes 57 (12), 3335–3343. 10.2337/db08-0503 18835934 PMC2584141

[B12] Chen Y. H. Lin H. Wang Q. Hou J. W. Mao Z. J. (2020). Protective role of silibinin against myocardial ischemia/reperfusion injury-induced cardiac dysfunction. Int. J. Biol. Sci. 16 (11), 1972–1988. 10.7150/ijbs.39259 32398964 PMC7211181

[B13] Chen T. Li K. Liu Z. Liu J. Wang Y. Sun R. (2021). WDR5 facilitates EMT and metastasis of CCA by increasing HIF-1α accumulation in Myc-dependent and independent pathways. Mol. Ther. 29 (6), 2134–2150. 10.1016/j.ymthe.2021.02.017 33601056 PMC8178527

[B14] Chen R. Zhang H. Tang B. Luo Y. Yang Y. Zhong X. (2024). Macrophages in cardiovascular diseases: molecular mechanisms and therapeutic targets. Signal Transduct. Target Ther. 9 (1), 130. 10.1038/s41392-024-01840-1 38816371 PMC11139930

[B15] Chen K. Q. Lei H. B. Liu X. Cao W. J. (2025). Mini-review: the health benefits and applications of allicin. Front. Pharmacol. 16, 1715922. 10.3389/fphar.2025.1715922 41383463 PMC12689917

[B16] Chi K. Yang S. Zhang Y. Zhao Y. Zhao J. Chen Q. (2024). Exploring the mechanism of Tingli Pill in the treatment of HFpEF based on network pharmacology and molecular docking. Med. Baltim. 103 (16), e37727. 10.1097/MD.0000000000037727 PMC11029988 38640300

[B17] Chinese Society of Cardiology (2025). Chinese guidelines for the diagnosis and treatment of heart failure 2024. Cardiol. Discov. 5 (1), 1–38. 10.1097/CD9.0000000000000146 PMC12059564 40351394

[B18] Choi J. Kim Y. Abeysiriwardhana H. N. I. Malla A. Anandappa J. M. (2025). Oxidative hypoxia drives TGF-β1-induced fibrosis under normoxia. Redox Biol. 89, 103947. 10.1016/j.redox.2025.103947 41344160 PMC12721191

[B19] Choya-Foces C. Navarro E. RíOS C. L. López M. G. Egea J. Hernansanz-Agustín P. (2024). The mitochondrial Na(+)/Ca(2+) exchanger NCLX is implied in the activation of hypoxia-inducible factors. Redox Biol. 77, 103364. 10.1016/j.redox.2024.103364 39341036 PMC11470253

[B20] Cluntun A. A. Badolia R. Lettlova S. Parnell K. M. Shankar T. S. Diakos N. A. (2021). The pyruvate-lactate axis modulates cardiac hypertrophy and heart failure. Cell. Metab. 33 (3), 629–648. 10.1016/j.cmet.2020.12.003 33333007 PMC7933116

[B21] Colturato C. P. Constantin R. P. Maeda A. S. JR. Yamamoto N. S. Bracht A. (2012). Metabolic effects of silibinin in the rat liver. Chem. Biol. Interact. 195 (2), 119–132. 10.1016/j.cbi.2011.11.006 22137898

[B22] Dai Z. Cheng J. Liu B. Yi D. Feng A. Wang T. (2021). Loss of endothelial hypoxia inducible factor-prolyl hydroxylase 2 induces cardiac hypertrophy and fibrosis. J. Am. Heart Assoc. 10 (22), e022077. 10.1161/JAHA.121.022077 34743552 PMC8751916

[B23] Dales J. P. Beaufils N. Silvy M. Picard C. Pauly V. Pradel V. (2010). Hypoxia inducible factor 1alpha gene (HIF-1alpha) splice variants: potential prognostic biomarkers in breast cancer. BMC Med. 8, 44. 10.1186/1741-7015-8-44 20624301 PMC2917392

[B24] Deberge M. Glinton K. Lantz C. Ge Z. D. Sullivan D. P. Patil S. (2025). Mechanical regulation of macrophage metabolism by allograft inflammatory factor 1 leal to adverse remodeling after cardiac injury. Nat. Cardiovasc Res. 4 (1), 83–101. 10.1038/s44161-024-00585-y 39747455 PMC12665373

[B25] Dengler V. L. Galbraith M. Espinosa J. M. (2014). Transcriptional regulation by hypoxia inducible factors. Crit. Rev. Biochem. Mol. Biol. 49 (1), 1–15. 10.3109/10409238.2013.838205 24099156 PMC4342852

[B26] Doedens A. L. Phan A. T. Stradner M. H. Fujimoto J. K. Nguyen J. V. Yang E. (2013). Hypoxia-inducible factors enhance the effector responses of CD8(+) T cells to persistent antigen. Nat. Immunol. 14 (11), 1173–1182. 10.1038/ni.2714 24076634 PMC3977965

[B27] Dong Z. Zhao P. Xu M. Zhang C. Guo W. Chen H. (2017). Astragaloside IV alleviates heart failure *via* activating PPARα to switch glycolysis to fatty acid β-oxidation. Sci. Rep. 7 (1), 2691. 10.1038/s41598-017-02360-5 28578382 PMC5457407

[B28] Drevytska T. Gonchar E. Okhai I. Lynnyk O. Mankovska I. Klionsky D. (2018). The protective effect of Hif3a RNA interference and HIF-prolyl hydroxylase inhibition on cardiomyocytes under anoxia-reoxygenation. Life Sci. 202, 131–139. 10.1016/j.lfs.2018.04.021 29660430

[B29] Dvorakova T. Finisguerra V. Formenti M. Loriot A. Boudhan L. Zhu J. (2024). Enhanced tumor response to adoptive T cell therapy with PHD2/3-deficient CD8 T cells. Nat. Commun. 15 (1), 7789. 10.1038/s41467-024-51782-z 39242595 PMC11379939

[B30] Eckle T. Hartmann K. Bonney S. Reithel S. Mittelbronn M. Walker L. A. (2012). Adora2b-elicited Per2 stabilization promotes a HIF-dependent metabolic switch crucial for myocardial adaptation to ischemia. Nat. Med. 18 (5), 774–782. 10.1038/nm.2728 22504483 PMC3378044

[B31] Eliceiri B. P. Paul R. Schwartzberg P. L. Hood J. D. Leng J. Cheresh D. A. (1999). Selective requirement for Src kinases during VEGF-induced angiogenesis and vascular permeability. Mol. Cell. 4 (6), 915–924. 10.1016/s1097-2765(00)80221-x 10635317

[B32] Eltzschig H. K. Bonney S. K. Eckle T. (2013). Attenuating myocardial ischemia by targeting A2B adenosine receptors. Trends Mol. Med. 19 (6), 345–354. 10.1016/j.molmed.2013.02.005 23540714 PMC3674126

[B33] Feng W. Ying Z. Ke F. Mei-Lin X. (2021). Apigenin suppresses TGF-β1-induced cardiac fibroblast differentiation and collagen synthesis through the downregulation of HIF-1α expression by miR-122-5p. Phytomedicine 83, 153481. 10.1016/j.phymed.2021.153481 33607460

[B34] Froese N. Szaroszyk M. Galuppo P. Visker J. R. Werlein C. Korf-Klingebiel M. (2024). Hypoxia attenuates pressure overload-induced heart failure. J. Am. Heart Assoc. 13 (3), e033553. 10.1161/JAHA.123.033553 38293923 PMC11056135

[B35] Fukuda R. Zhang H. Kim J. W. Shimoda L. Dang C. V. Semenza G. L. (2007). HIF-1 regulates cytochrome oxidase subunits to optimize efficiency of respiration in hypoxic cells. Cell. 129 (1), 111–122. 10.1016/j.cell.2007.01.047 17418790

[B36] Gibb A. A. Hill B. G. (2018). Metabolic coordination of physiological and pathological cardiac remodeling. Circ. Res. 123 (1), 107–128. 10.1161/CIRCRESAHA.118.312017 29929976 PMC6023588

[B37] Gong Y. T. Li Y. P. Cheng Y. R. Shi X. J. Yang L. Yang D. P. (2021). Molecular mechanism of Qishen Yiqi dripping pills in treating myocardial ischemia: a study based on HIF-1 signaling pathway. Zhongguo Zhong Yao Za Zhi 46 (15), 3949–3959. 10.19540/j.cnki.cjcmm.20210524.702 34472272

[B38] Gu M. Singh R. P. Dhanalakshmi S. Agarwal C. Agarwal R. (2007). Silibinin inhibits inflammatory and angiogenic attributes in photocarcinogenesis in SKH-1 hairless mice. Cancer Res. 67 (7), 3483–3491. 10.1158/0008-5472.CAN-06-3955 17409458

[B39] Guo J. Mihic A. Wu J. Zhang Y. Singh K. Dhingra S. (2015). Canopy 2 attenuates the transition from compensatory hypertrophy to dilated heart failure in hypertrophic cardiomyopathy. Eur. Heart J. 36 (37), 2530–2540. 10.1093/eurheartj/ehv294 26160001 PMC4589657

[B40] He X. Cantrell A. C. Williams Q. A. Gu W. Chen Y. Chen J. X. (2024). p53 acetylation exerts critical roles in pressure overload-induced coronary microvascular dysfunction and heart failure in mice. Arterioscler. Thromb. Vasc. Biol. 44 (4), 826–842. 10.1161/ATVBAHA.123.319601 38328937 PMC10978286

[B41] Heidenreich P. A. Bozkurt B. Aguilar D. Allen L. A. Byun J. J. Colvin M. M. (2022). 2022 AHA/ACC/HFSA guideline for the management of heart failure: a report of the American college of cardiology/American heart association joint committee on clinical practice guidelines. Circulation 145 (18), e895–e1032. 10.1161/CIR.0000000000001063 35363499

[B42] Heikkilä M. Pasanen A. Kivirikko K. I. Myllyharju J. (2011). Roles of the human hypoxia-inducible factor (HIF)-3α variants in the hypoxia response. Cell. Mol. Life Sci. 68 (23), 3885–3901. 10.1007/s00018-011-0679-5 21479871 PMC11114783

[B43] Hinton A. JR. Claypool S. M. Neikirk K. Senoo N. Wanjalla C. N. Kirabo A. (2024). Mitochondrial structure and function in human heart failure. Circ. Res. 135 (2), 372–396. 10.1161/CIRCRESAHA.124.323800 38963864 PMC11225798

[B44] Hoefflin R. Harlander S. SchäFER S. Metzger P. Kuo F. Schönenberger D. (2020). HIF-1α and HIF-2α differently regulate tumour development and inflammation of clear cell renal cell carcinoma in mice. Nat. Commun. 11 (1), 4111. 10.1038/s41467-020-17873-3 32807776 PMC7431415

[B45] HöLSCHER M. SchäFER K. Krull S. Farhat K. Hesse A. Silter M. (2012). Unfavourable consequences of chronic cardiac HIF-1α stabilization. Cardiovasc Res. 94 (1), 77–86. 10.1093/cvr/cvs014 22258630

[B46] Huang M. Nguyen P. Jia F. Hu S. Gong Y. de Almeida P. E. (2011). Double knockdown of prolyl hydroxylase and factor-inhibiting hypoxia-inducible factor with nonviral minicircle gene therapy enhances stem cell mobilization and angiogenesis after myocardial infarction. Circulation 124 (11 Suppl. l), S46–S54. 10.1161/CIRCULATIONAHA.110.014019 21911818 PMC3181087

[B47] Huang J. Trych U. Marszałek K. (2026). Unraveling health benefits of garlic (Allium sativum L.): investigating the influence of processing methods. Compr. Rev. Food Sci. Food Saf. 25 (1), e70357. 10.1111/1541-4337.70357 41414732

[B48] Hwang H. J. Lynn S. G. Vengellur A. Saini Y. Grier E. A. Ferguson-Miller S. M. (2015). Hypoxia inducible factors modulate mitochondrial oxygen consumption and transcriptional regulation of nuclear-encoded electron transport chain genes. Biochemistry 54 (24), 3739–3748. 10.1021/bi5012892 26030260 PMC5957085

[B49] Jaskiewicz M. Moszynska A. Serocki M. Króliczewski J. Bartoszewska S. Collawn J. F. (2022). Hypoxia-inducible factor (HIF)-3a2 serves as an endothelial cell fate executor during chronic hypoxia. Excli J. 21, 454–469. 10.17179/excli2021-4622 35391921 PMC8983852

[B50] Kashihara T. Mukai R. Oka S. I. Zhai P. Nakada Y. Yang Z. (2022). YAP mediates compensatory cardiac hypertrophy through aerobic glycolysis in response to pressure overload. J. Clin. Invest. 132 (6), e150595. 10.1172/JCI150595 35133975 PMC8920343

[B51] Khalil N. N. Rexius-Hall M. L. Escopete S. Parker S. J. McCain M. L. (2024). Distinct phenotypes induced by acute hypoxia and TGF-β1 in human adult cardiac fibroblasts. J. Mol. Cell. Cardiol. Plus 9, 100080. 10.1016/j.jmccpl.2024.100080 39329164 PMC11423773

[B52] Khan M. S. Solomon N. Devore A. D. Sharma A. Felker G. M. Hernandez A. F. (2022). Clinical outcomes with metformin and sulfonylurea therapies among patients with heart failure and diabetes. JACC Heart Fail 10 (3), 198–210. 10.1016/j.jchf.2021.11.001 34895861

[B53] Khan M. S. Shahid I. Bennis A. Rakisheva A. Metra M. Butler J. (2024). Global epidemiology of heart failure. Nat. Rev. Cardiol. 21 (10), 717–734. 10.1038/s41569-024-01046-6 38926611

[B54] Kim J. W. Tchernyshyov I. Semenza G. L. Dang C. V. (2006). HIF-1-mediated expression of pyruvate dehydrogenase kinase: a metabolic switch required for cellular adaptation to hypoxia. Cell. Metab. 3 (3), 177–185. 10.1016/j.cmet.2006.02.002 16517405

[B55] Koh M. Y. Powis G. (2012). Passing the baton: the HIF switch. Trends Biochem. Sci. 37 (9), 364–372. 10.1016/j.tibs.2012.06.004 22818162 PMC3433036

[B56] Krishnan J. Suter M. Windak R. Krebs T. Felley A. Montessuit C. (2009). Activation of a HIF1alpha-PPARgamma axis underlies the integration of glycolytic and lipid anabolic pathways in pathologic cardiac hypertrophy. Cell. Metab. 9 (6), 512–524. 10.1016/j.cmet.2009.05.005 19490906

[B57] Kumar A. A. Kelly D. P. Chirinos J. A. (2019). Mitochondrial dysfunction in heart failure with preserved ejection fraction. Circulation 139 (11), 1435–1450. 10.1161/CIRCULATIONAHA.118.036259 30856000 PMC6414077

[B58] Lando D. Peet D. J. Gorman J. J. Whelan D. A. Whitelaw M. L. Bruick R. K. (2002). FIH-1 is an asparaginyl hydroxylase enzyme that regulates the transcriptional activity of hypoxia-inducible factor. Genes. Dev. 16 (12), 1466–1471. 10.1101/gad.991402 12080085 PMC186346

[B59] Li Y. Yan C. Fan J. Hou Z. Han Y. (2021). MiR-221-3p targets Hif-1α to inhibit angiogenesis in heart failure. Lab. Invest. 101 (1), 104–115. 10.1038/s41374-020-0450-3 32873879

[B60] Li Z. Shen L. Tu Y. Lu S. Liu B. (2025a). Hypoxia-inducible factor-prolyl hydroxylase inhibitors in treatment of anemia with chronic disease. Chin. Med. J. Engl. 138 (12), 1424–1432. 10.1097/CM9.0000000000003470 40405347 PMC12180824

[B61] Li Q. Zhang S. Li Y. Yao N. Feng Y. Yang G. (2025b). Astragaloside IV alleviates radiation-induced heart disease by regulating energy metabolism. Phytomedicine 146, 157135. 10.1016/j.phymed.2025.157135 40774010

[B62] Li G. Wang M. Dong Q. Li D. Liu J. Long Q. (2025c). Astragaloside IV ameliorates cardiomyocyte injury and heart failure through hif/rho/rock pathway regulation: *in vitro* and *in vivo* insights. J. Bioenerg. Biomembr. 57 (6), 351–364. 10.1007/s10863-025-10073-y 41085879

[B63] Li J. Jiao B. Wang K. Jiao S. Wang R. Sun Y. (2025d). Draconis sanguis (DS) from the fruit of Daemonorops draco Bl. ameliorates cardiac function through optimizing myocardial energy metabolism by promoting angiogenesis in ischemic heart failure. Phytomedicine 140, 156583. 10.1016/j.phymed.2025.156583 40085987

[B64] Li X. Li D. Liu S. Wang Y. Fan X. Yan Z. (2025e). Shenfu injection inhibits myocardial fibrosis by regulating glycolysis through the PI3K-AKT/HIF-1α signaling pathway. Phytomedicine 149, 157529. 10.1016/j.phymed.2025.157529 41273867

[B65] Liang N. Liu J. H. Li Q. He Z. Huang X. Chen Z. Y. (2025). Roles of garlic and its bioactive compounds in modulating cholesterol metabolism. Phytomedicine 149, 157543. 10.1016/j.phymed.2025.157543 41273868

[B66] Lim B. Jung K. Gwon Y. Oh J. G. Roh J. I. Hong S. H. (2019). Cardioprotective role of APIP in myocardial infarction through ADORA2B. Cell. Death Dis. 10 (7), 511. 10.1038/s41419-019-1746-3 31263105 PMC6602929

[B67] Lin J. Wang Q. Hua X. Duan J. Yao K. (2022). Integrated gut-heart axis and network pharmacology to reveal the mechanisms of the huoxue wentong formula against myocardial ischemia. Evid. Based Complement. Altern. Med. 2022, 9538512. 10.1155/2022/9538512 PMC9117028 35600966

[B68] Lin Z. J. Dong X. He H. Jiang J. L. Guan Z. J. Li X. (2024). A simplified herbal decoction attenuates myocardial infarction by regulating macrophage metabolic reprogramming and phenotypic differentiation via modulation of the HIF-1α/PDK1 axis. Chin. Med. 19 (1), 75. 10.1186/s13020-024-00933-x 38816815 PMC11140944

[B69] Lisy K. Peet D. J. (2008). Turn me on: regulating HIF transcriptional activity. Cell. Death Differ. 15 (4), 642–649. 10.1038/sj.cdd.4402315 18202699

[B70] Liu Y. Zhang Q. Yang L. Tian W. Yang Y. Xie Y. (2022a). Metformin attenuates cardiac hypertrophy via the HIF-1α/PPAR-γ signaling pathway in high-fat diet rats. Front. Pharmacol. 13, 919202. 10.3389/fphar.2022.919202 35833024 PMC9271627

[B71] Liu T. Yan T. Jia X. Liu J. Wang Y. (2022b). Systematic exploration of the potential material basis and molecular mechanism of the Mongolian medicine Nutmeg-5 in improving cardiac remodeling after myocardial infarction. J. Ethnopharmacol. 285, 114847. 10.1016/j.jep.2021.114847 34800647

[B72] López-Mejía A. Briseño-Díaz P. Robles-Flores M. (2025). The role of hypoxia-inducible factor-3α in human disease. Biochim. Biophys. Acta Mol. Cell. Res. 1872 (7), 120007. 10.1016/j.bbamcr.2025.120007 40513617

[B73] Lunderberg J. M. Spicer A. J. Cassavaugh J. Jalkanen J. Haskó G. Robson S. C. (2025). Targeting CD73 and correcting adenosinergic signaling in critically ill patients. Front. Pharmacol. 16, 1601481. 10.3389/fphar.2025.1601481 41601962 PMC12832292

[B74] Makino Y. Kanopka A. Wilson W. J. Tanaka H. Poellinger L. (2002). Inhibitory PAS domain protein (IPAS) is a hypoxia-inducible splicing variant of the hypoxia-inducible factor-3alpha locus. J. Biol. Chem. 277 (36), 32405–32408. 10.1074/jbc.C200328200 12119283

[B75] Makino Y. Uenishi R. Okamoto K. Isoe T. Hosono O. Tanaka H. (2007). Transcriptional up-regulation of inhibitory PAS domain protein gene expression by hypoxia-inducible factor 1 (HIF-1): a negative feedback regulatory circuit in HIF-1-mediated signaling in hypoxic cells. J. Biol. Chem. 282 (19), 14073–14082. 10.1074/jbc.M700732200 17355974

[B76] Maynard M. A. Evans A. J. Hosomi T. Hara S. Jewett M. A. S. Ohh M. (2005). Human HIF-3alpha4 is a dominant-negative regulator of HIF-1 and is down-regulated in renal cell carcinoma. Faseb J. 19 (11), 1396–1406. 10.1096/fj.05-3788com 16126907

[B77] Maynard M. A. Evans A. J. Shi W. Kim W. Y. Liu F. F. Ohh M. (2007). Dominant-negative HIF-3 alpha 4 suppresses VHL-null renal cell carcinoma progression. Cell. Cycle 6 (22), 2810–2816. 10.4161/cc.6.22.4947 17998805

[B78] Mcdonagh T. A. Metra M. Adamo M. Gardner R. S. Baumbach A. Böhm M. (2021). ESC guidelines for the diagnosis and treatment of acute and chronic heart failure. Eur. Heart J. 42 (36), 3599–3726. 10.1093/eurheartj/ehab368 34447992

[B79] Mcdonagh T. A. Metra M. Adamo M. Gardner R. S. Baumbach A. Böhm M. (2023). Focused update of the 2021 ESC guidelines for the diagnosis and treatment of acute and chronic heart failure. Eur. Heart J. 44 (37), 3627–3639. 10.1093/eurheartj/ehad195 37622666

[B80] Mcgettrick A. F. O'Neill L. A. J. (2020). The role of HIF in immunity and inflammation. Cell. Metab. 32 (4), 524–536. 10.1016/j.cmet.2020.08.002 32853548

[B81] Mialet-Perez J. Belaidi E. (2024). Interplay between hypoxia inducible Factor-1 and mitochondria in cardiac diseases. Free Radic. Biol. Med. 221, 13–22. 10.1016/j.freeradbiomed.2024.04.239 38697490

[B82] Mohammed S. F. Hussain S. Mirzoyev S. A. Edwards W. D. Maleszewski J. J. Redfield M. M. (2015). Coronary microvascular rarefaction and myocardial fibrosis in heart failure with preserved ejection fraction. Circulation 131 (6), 550–559. 10.1161/CIRCULATIONAHA.114.009625 25552356 PMC4324362

[B83] Montaigne D. Butruille L. Staels B. (2021). PPAR control of metabolism and cardiovascular functions. Nat. Rev. Cardiol. 18 (12), 809–823. 10.1038/s41569-021-00569-6 34127848

[B84] Najjar R. S. Roy R. K. Stern J. E. Feresin R. G. (2024). Raspberry polyphenols target molecular pathways of heart failure. J. Nutr. Biochem. 124, 109535. 10.1016/j.jnutbio.2023.109535 37984734 PMC12862629

[B85] Nakanishi T. Kuragano T. (2024). Growing concerns about using hypoxia-inducible factor prolyl hydroxylase inhibitors for the treatment of renal anemia. Clin. Kidney J. 17 (3), sfae051. 10.1093/ckj/sfae051 38516524 PMC10956400

[B86] Oka T. Akazawa H. Naito A. T. Komuro I. (2014). Angiogenesis and cardiac hypertrophy: maintenance of cardiac function and causative roles in heart failure. Circ. Res. 114 (3), 565–571. 10.1161/CIRCRESAHA.114.300507 24481846

[B87] Ouyang J. Lian K. Liao X. Meng L. Li L. Zhao Z. (2025). Shenfu injection ameliorates chronic heart failure by regulating glycolysis mediated by the HIF-1α/PFKFB3 pathway. Chin. J. Exp. Tradit. Med. Formulae 31 (16), 136–145. 10.13422/j.cnki.syfjx.20250615

[B88] Oyama Y. Bartman C. M. Bonney S. Lee J. S. Walker L. A. Han J. (2019). Intense light-mediated circadian cardioprotection *via* transcriptional reprogramming of the endothelium. Cell. Rep. 28 (6), 1471–1484. 10.1016/j.celrep.2019.07.020 31390562 PMC6708043

[B89] Packer M. (2020). Mutual antagonism of hypoxia-inducible factor isoforms in cardiac, vascular, and renal disorders. JACC Basic Transl. Sci. 5 (9), 961–968. 10.1016/j.jacbts.2020.05.006 33015417 PMC7524787

[B90] Pan L. He X. Xu R. Bhattarai U. Wang D. Wang H. (2025). PHD2 deletion in CD8(+) T cells worsens TAC-Induced cardiac inflammation, heart failure, and pulmonary remodeling. Hypertension 82 (11), 2040–2054. 10.1161/HYPERTENSIONAHA.125.25284 40959903 PMC12747131

[B91] Papandreou I. Cairns R. A. Fontana L. Lim A. L. Denko N. C. (2006). HIF-1 mediates adaptation to hypoxia by actively downregulating mitochondrial oxygen consumption. Cell. Metab. 3 (3), 187–197. 10.1016/j.cmet.2006.01.012 16517406

[B92] Parmley W. W. (1989). Pathophysiology and current therapy of congestive heart failure. J. Am. Coll. Cardiol. 13 (4), 771–785. 10.1016/0735-1097(89)90215-5 2647811

[B93] Pasanen A. Heikkilä M. Rautavuoma K. Hirsilä M. Kivirikko K. I. Myllyharju J. (2010). Hypoxia-inducible factor (HIF)-3alpha is subject to extensive alternative splicing in human tissues and cancer cells and is regulated by HIF-1 but not HIF-2. Int. J. Biochem. Cell. Biol. 42 (7), 1189–1200. 10.1016/j.biocel.2010.04.008 20416395

[B94] Qiu B. Qiao S. Shi X. Deng B. Ma Z. (2024). Shen’ge formula protects cardiac function in rats with pressure overload-induced heart failure. Drug Des. Devel Ther. 18, 1875–1890. 10.2147/DDDT.S451720 38831869 PMC11146625

[B95] Reiter R. Swingen C. Moore L. Henry T. D. Traverse J. H. (2012). Circadian dependence of infarct size and left ventricular function after ST elevation myocardial infarction. Circ. Res. 110 (1), 105–110. 10.1161/CIRCRESAHA.111.254284 22095727 PMC3253266

[B96] Ren H. Wang S. S. Zhao W. Z. Xu S. H. Wei K. D. Wu W. W. (2024). Bioinformatics and animal experiments reveal mechanism of Linggui Zhugan decoction in ameliorating chronic heart failure after myocardial infarction via HIF-1α/HO-1 signaling pathway. Zhongguo Zhong Yao Za Zhi 49 (23), 6407–6416. 10.19540/j.cnki.cjcmm.20240718.401 39805787

[B97] Riley P. R. Smart N. (2011). Vascularizing the heart. Cardiovasc Res. 91 (2), 260–268. 10.1093/cvr/cvr035 21282300

[B98] Ritterhoff J. Tian R. (2017). Metabolism in cardiomyopathy: every substrate matters. Cardiovasc Res. 113 (4), 411–421. 10.1093/cvr/cvx017 28395011 PMC5852620

[B99] Rodriguez J. Herrero A. Li S. Rauch N. Quintanilla A. Wynne K. (2018). PHD3 regulates p53 protein stability by hydroxylating proline 359. Cell. Rep. 24 (5), 1316–1329. 10.1016/j.celrep.2018.06.108 30067985 PMC6088137

[B100] Sánchez-Gloria J. L. Martínez-Olivares C. E. Del Valle-Mondragón L. Cortes-Camacho F. Zambrano-Vasquez O. R. Hernandez-Pando R. (2023). Allicin, an emerging treatment for pulmonary arterial hypertension: an experimental study. Int. J. Mol. Sci. 24 (16), 12959. 10.3390/ijms241612959 37629140 PMC10454707

[B101] Sano M. Minamino T. Toko H. Miyauchi H. Orimo M. Qin Y. (2007). p53-induced inhibition of Hif-1 causes cardiac dysfunction during pressure overload. Nature 446 (7134), 444–448. 10.1038/nature05602 17334357

[B102] Sant'Ana P. G. Tomasi L. C. Murata G. M. Vileigas D. F. Ferreira Mota G. A. de Souza S. L. B. (2023). Hypoxia-inducible factor 1-Alpha and glucose metabolism during cardiac remodeling progression from hypertrophy to heart failure. Int. J. Mol. Sci. 24 (7), 6201. 10.3390/ijms24076201 37047174 PMC10094437

[B103] Santos-Zas I. Lemarié J. Zlatanova I. Cachanado M. Seghezzi J. C. Benamer H. (2021). Cytotoxic CD8(+) T cells promote granzyme B-dependent adverse post-ischemic cardiac remodeling. Nat. Commun. 12 (1), 1483. 10.1038/s41467-021-21737-9 33674611 PMC7935973

[B104] Savarese G. Becher P. M. Lund L. H. Seferovic P. Rosano G. M. C. Coats A. J. S. (2023). Global burden of heart failure: a comprehensive and updated review of epidemiology. Cardiovasc Res. 118 (17), 3272–3287. 10.1093/cvr/cvac013 35150240

[B105] SchöDEL J. Oikonomopoulos S. Ragoussis J. Pugh C. W. Ratcliffe P. J. Mole D. R. (2011). High-resolution genome-wide mapping of HIF-binding sites by ChIP-seq. Blood 117 (23), e207–e217. 10.1182/blood-2010-10-314427 21447827 PMC3374576

[B106] Schönenberger M. J. Krek W. Kovacs W. J. (2015). EPAS1/HIF-2α is a driver of mammalian pexophagy. Autophagy 11 (6), 967–969. 10.1080/15548627.2015.1045180 25997392 PMC4502715

[B107] Sellam L. S. Zappasodi R. Chettibi F. Djennaoui D. Yahi-Ait Mesbah N. Amir-Tidadini Z. C. (2020). Silibinin down-regulates PD-L1 expression in nasopharyngeal carcinoma by interfering with tumor cell glycolytic metabolism. Arch. Biochem. Biophys. 690, 108479. 10.1016/j.abb.2020.108479 32679194 PMC8507490

[B108] Semenza G. L. (2014a). Oxygen sensing, hypoxia-inducible factors, and disease pathophysiology. Annu. Rev. Pathol. 9, 47–71. 10.1146/annurev-pathol-012513-104720 23937437

[B109] Semenza G. L. (2014b). Hypoxia-inducible factor 1 and cardiovascular disease. Annu. Rev. Physiol. 76, 39–56. 10.1146/annurev-physiol-021113-170322 23988176 PMC4696033

[B110] Singh R. P. Gu M. Agarwal R. (2008). Silibinin inhibits colorectal cancer growth by inhibiting tumor cell proliferation and angiogenesis. Cancer Res. 68 (6), 2043–2050. 10.1158/0008-5472.CAN-07-6247 18339887

[B111] Skuli N. Majmundar A. J. Krock B. L. Mesquita R. C. Mathew L. K. Quinn Z. L. (2012). Endothelial HIF-2α regulates murine pathological angiogenesis and revascularization processes. J. Clin. Invest. 122 (4), 1427–1443. 10.1172/JCI57322 22426208 PMC3314446

[B112] Sole M. J. Martino T. A. (2023). Circadian medicine: a critical strategy for cardiac care. Nat. Rev. Cardiol. 20 (11), 715–716. 10.1038/s41569-023-00925-8 37644115

[B113] Fialho M. L. S. Abd Jamil A. H. Stannard G. A. Heather L. C. (2019). Hypoxia-inducible factor 1 signalling, metabolism and its therapeutic potential in cardiovascular disease. Biochim. Biophys. Acta Mol. Basis Dis. 1865 (4), 831–843. 10.1016/j.bbadis.2018.09.024 30266651

[B114] Su Z. Liu Y. Zhang H. (2021). Adaptive cardiac metabolism under chronic hypoxia: mechanism and clinical implications. Front. Cell. Dev. Biol. 9, 625524. 10.3389/fcell.2021.625524 33604337 PMC7884626

[B115] Sui Y. B. Wang Y. Liu L. Liu F. Zhang Y. Q. (2019). Astragaloside IV alleviates heart failure by promoting angiogenesis through the JAK-STAT3 pathway. Pharm. Biol. 57 (1), 48–54. 10.1080/13880209.2019.1569697 30905241 PMC8871603

[B116] Sun X. Q. Li X. Li Y. Q. Lu X. Y. Liu X. N. Cui L. W. (2025). Qishen granules modulate metabolism flexibility against myocardial infarction *via* HIF-1 α-Dependent mechanisms in rats. Chin. J. Integr. Med. 31 (3), 215–227. 10.1007/s11655-024-3667-y 39305458

[B117] Takeda N. O’Dea E. L. Doedens A. Kim J. W. Weidemann A. Stockmann C. (2010). Differential activation and antagonistic function of HIF-{alpha} isoforms in macrophages are essential for NO homeostasis. Genes. Dev. 24 (5), 491–501. 10.1101/gad.1881410 20194441 PMC2827844

[B118] Tanaka T. Wiesener M. Bernhardt W. Eckardt K. U. Warnecke C. (2009). The human HIF (hypoxia-inducible factor)-3alpha gene is a HIF-1 target gene and may modulate hypoxic gene induction. Biochem. J. 424 (1), 143–151. 10.1042/BJ20090120 19694616

[B119] Tang S. Fan C. Iroegbu C. D. Zhou W. Zhang Z. Wu M. (2021). TMSB4 overexpression enhances the potency of marrow mesenchymal stromal cells for myocardial repair. Front. Cell. Dev. Biol. 9, 670913. 10.3389/fcell.2021.670913 34178995 PMC8221609

[B120] Tello D. Balsa E. Acosta-Iborra B. Fuertes-Yebra E. Elorza A. Ordóñez Á. (2011). Induction of the mitochondrial NDUFA4L2 protein by HIF-1α decreases oxygen consumption by inhibiting complex I activity. Cell. Metab. 14 (6), 768–779. 10.1016/j.cmet.2011.10.008 22100406

[B121] Thorp E. B. Filipp M. (2025). Contributions of inflammation to cardiometabolic heart failure with preserved ejection fraction. Annu. Rev. Pathol. 20 (1), 143–167. 10.1146/annurev-pathmechdis-111523-023405 39357068 PMC12925995

[B122] Thosar S. S. Butler M. P. Shea S. A. (2018). Role of the circadian system in cardiovascular disease. J. Clin. Invest. 128 (6), 2157–2167. 10.1172/JCI80590 29856365 PMC5983320

[B123] Tolonen J. P. Heikkilä M. Malinen M. Lee H. M. Palvimo J. J. Wei G. H. (2020). A long hypoxia-inducible factor 3 isoform 2 is a transcription activator that regulates erythropoietin. Cell. Mol. Life Sci. 77 (18), 3627–3642. 10.1007/s00018-019-03387-9 31768607 PMC7452874

[B124] Torii S. Goto Y. Ishizawa T. Hoshi H. Goryo K. Yasumoto K. (2011). Pro-apoptotic activity of inhibitory PAS domain protein (IPAS), a negative regulator of HIF-1, through binding to pro-survival Bcl-2 family proteins. Cell. Death Differ. 18 (11), 1711–1725. 10.1038/cdd.2011.47 21546903 PMC3190112

[B125] Ullah K. Ai L. Li Y. Liu L. Zhang Q. Pan K. (2025). ARNT-dependent HIF-2α signaling protects cardiac microvascular barrier integrity and heart function post-myocardial infarction. Commun. Biol. 8 (1), 440. 10.1038/s42003-025-07753-1 40089572 PMC11910586

[B126] Villareal L. B. Falcon D. M. Xie L. Xue X. (2024). Hypoxia-inducible factor 3α1 increases epithelial-to-mesenchymal transition and iron uptake to drive colorectal cancer liver metastasis. Br. J. Cancer 130 (12), 1904–1915. 10.1038/s41416-024-02699-3 38693428 PMC11183190

[B127] Walter K. M. Schönenberger M. J. Trötzmüller M. Horn M. Elsässer H. P. Moser A. B. (2014). Hif-2α promotes degradation of mammalian peroxisomes by selective autophagy. Cell. Metab. 20 (5), 882–897. 10.1016/j.cmet.2014.09.017 25440060

[B128] Wan J. Zhang Z. Wu C. Tian S. Zang Y. Jin G. (2023). Astragaloside IV derivative HHQ16 ameliorates infarction-induced hypertrophy and heart failure through degradation of lncRNA4012/9456. Signal Transduct. Target Ther. 8 (1), 414. 10.1038/s41392-023-01660-9 37857609 PMC10587311

[B129] Wang G. L. Jiang B. H. Rue E. A. Semenza G. L. (1995). Hypoxia-inducible factor 1 is a basic-helix-loop-helix-PAS heterodimer regulated by cellular O2 tension. Proc. Natl. Acad. Sci. U. S. A. 92 (12), 5510–5514. 10.1073/pnas.92.12.5510 7539918 PMC41725

[B130] Wang J. Duan Y. Sluijter J. P. Xiao J. (2019). Lymphocytic subsets play distinct roles in heart diseases. Theranostics 9 (14), 4030–4046. 10.7150/thno.33112 31281530 PMC6592175

[B131] Wang Y. Fu M. Wang J. Zhang J. Han X. Song Y. (2020). Qiliqiangxin improves cardiac function through regulating energy metabolism via HIF-1α-Dependent and independent mechanisms in heart failure rats after acute myocardial infarction. Biomed. Res. Int. 2020, 1276195. 10.1155/2020/1276195 32626732 PMC7306086

[B132] Wang H. Chai K. Du M. Wang S. Cai J. P. Li Y. (2021). Prevalence and incidence of heart failure among urban patients in China: a national population-based analysis. Circ. Heart Fail 14 (10), e008406. 10.1161/CIRCHEARTFAILURE.121.008406 34455858

[B133] Wang H. Zhou H. Yan S. Liu Y. Kuhn M. Prettner K. (2025a). Heart failure in China: a macroeconomic modelling study of intervention strategies. Eur. Heart J. 47 (8), 974–984. 10.1093/eurheartj/ehaf992 41355346

[B134] Wang M. Chen J. Zhang Z. Wang T. Zhao J. Wang X. (2025b). Silybin mitigates post-myocardial infarction heart failure in mice via modulation of HIF-1α-Driven glycolysis and energy metabolism. Nutrients 17 (17), 2800. 10.3390/nu17172800 40944191 PMC12430516

[B135] Wang P. Zhang X. P. Liu F. Wang W. (2025c). Progressive deactivation of hydroxylases controls hypoxia-inducible Factor-1α-Coordinated cellular adaptation to graded hypoxia. Res. (Washington DC) 8, 0651. 10.34133/research.0651 PMC11960303 40171017

[B136] Wang X. Zhao M. Xue W. Wang J. Pang X. Zheng D. (2026). (2S)-5-methoxy-6-methyl-flavan-7-ol from Sanguis draconis attenuates atherosclerosis in ApoE (-/-) mice by inhibiting monocyte-endothelial cell adhesion. J. Ethnopharmacol. 356, 120823. 10.1016/j.jep.2025.120823 41161624

[B137] Willam C. Maxwell P. H. Nichols L. Lygate C. Tian Y. M. Bernhardt W. (2006). HIF prolyl hydroxylases in the rat; organ distribution and changes in expression following hypoxia and coronary artery ligation. J. Mol. Cell. Cardiol. 41 (1), 68–77. 10.1016/j.yjmcc.2006.04.009 16765982

[B138] Wright C. W. Duckett C. S. (2009). The aryl hydrocarbon nuclear translocator alters CD30-mediated NF-kappaB-dependent transcription. Science 323 (5911), 251–255. 10.1126/science.1162818 19131627 PMC2682336

[B139] Wu R. Chang H. C. Khechaduri A. Chawla K. Tran M. Chai X. (2014). Cardiac-specific ablation of ARNT leads to lipotoxicity and cardiomyopathy. J. Clin. Invest. 124 (11), 4795–4806. 10.1172/JCI76737 25329697 PMC4347233

[B140] Wu X. Reboll M. R. Korf-Klingebiel M. Wollert K. C. (2021a). Angiogenesis after acute myocardial infarction. Cardiovasc Res. 117 (5), 1257–1273. 10.1093/cvr/cvaa287 33063086

[B141] Wu Y. Li Z. Mcdonough M. A. Schofield C. J. Zhang X. (2021b). Inhibition of the oxygen-sensing asparaginyl hydroxylase factor inhibiting hypoxia-inducible factor: a potential hypoxia response modulating strategy. J. Med. Chem. 64 (11), 7189–7209. 10.1021/acs.jmedchem.1c00415 34029087

[B142] Xia X. Lemieux M. E. Li W. Carroll J. S. Brown M. Liu X. S. (2009). Integrative analysis of HIF binding and transactivation reveals its role in maintaining histone methylation homeostasis. Proc. Natl. Acad. Sci. U. S. A. 106 (11), 4260–4265. 10.1073/pnas.0810067106 19255431 PMC2657396

[B143] Xu X. Zhen P. H. Yu F. C. Wang T. Li S. N. Wei Q. (2022). Chronic intermittent hypoxia accelerates cardiac dysfunction and cardiac remodeling during cardiac pressure overload in mice and can be alleviated by PHD3 overexpression. Front. Cardiovasc Med. 9, 974345. 10.3389/fcvm.2022.974345 36172572 PMC9510693

[B144] Xu L. Yang M. Wei A. Wei Z. Qin Y. Wang K. (2024). Aerobic exercise-induced HIF-1α upregulation in heart failure: exploring potential impacts on MCT1 and MPC1 regulation. Mol. Med. 30 (1), 83. 10.1186/s10020-024-00854-3 38867145 PMC11167843

[B145] Xue X. Jungles K. Onder G. Samhoun J. Győrffy B. Hardiman K. M. (2016). HIF-3α1 promotes colorectal tumor cell growth by activation of JAK-STAT3 signaling. Oncotarget 7 (10), 11567–11579. 10.18632/oncotarget.7272 26871465 PMC4905494

[B146] Yamashita T. Ohneda O. Nagano M. Iemitsu M. Makino Y. Tanaka H. (2008). Abnormal heart development and lung remodeling in mice lacking the hypoxia-inducible factor-related basic helix-loop-helix PAS protein NEPAS. Mol. Cell Biol. 28 (4), 1285–1297. 10.1128/MCB.01332-07 18070924 PMC2258751

[B147] Yoshitake T. Hashimoto T. Matsushima S. Ikuta K. Yamamoto S. Suenaga T. (2025). Effects of hypoxia-inducible factor prolyl hydroxylase inhibitors on relationship between B-Type natriuretic peptide and hemoglobin levels in patients with cardiorenal anemia syndrome. Cardiovasc Ther. 2025, 3143864. 10.1155/cdr/3143864 41244933 PMC12615038

[B148] Zhang L. Jain M. K. (2021). Circadian regulation of cardiac metabolism. J. Clin. Invest. 131 (15), e148276. 10.1172/JCI148276 34338224 PMC8321567

[B149] Zhang P. Bai Y. Lu L. Li Y. Duan C. (2016). An oxygen-insensitive Hif-3α isoform inhibits Wnt signaling by destabilizing the nuclear β-catenin complex. Elife 5, e08996. 10.7554/eLife.08996 PMC4769163 26765566

[B150] Zhang Q. Guo D. Wang Y. Wang X. Wang Q. Wu Y. (2020). Danqi pill protects against heart failure post-acute myocardial infarction via HIF-1α/PGC-1α mediated glucose metabolism pathway. Front. Pharmacol. 11, 458. 10.3389/fphar.2020.00458 32372956 PMC7187888

[B151] Zhao Y. M. Wang X. Y. Pei Y. J. Xue W. G. Wang J. J. Pang X. P. (2024). Effect and mechanism of EtOAc extract of Draconis Sanguis on improving atherosclerosis in ApoE∼(-/-) mice. Zhongguo Zhong Yao Za Zhi 49 (11), 2973–2980. 10.19540/j.cnki.cjcmm.20240122.403 39041157

[B152] Zhao Y. J. Wu W. H. Niu K. M. Zhang W. J. Li S. R. Bao R. L. (2025). Xinkeshu formula restrains pathological cardiac hypertrophy through metabolic remodeling via AMPK/mTOR pathway. Phytomedicine 136, 156309. 10.1016/j.phymed.2024.156309 39700635

