# Supplementary figures and images for: HIF-mediated hierarchical hypoxic adaptation, a novel paradigm in heart failure pathogenesis: is there a role for targeted therapies?
Source: Front Pharmacol. 2026 Mar 16;17:1771529. doi: 10.3389/fphar.2026.1771529 (PMC13033609; doi:10.3389/fphar.2026.1771529)

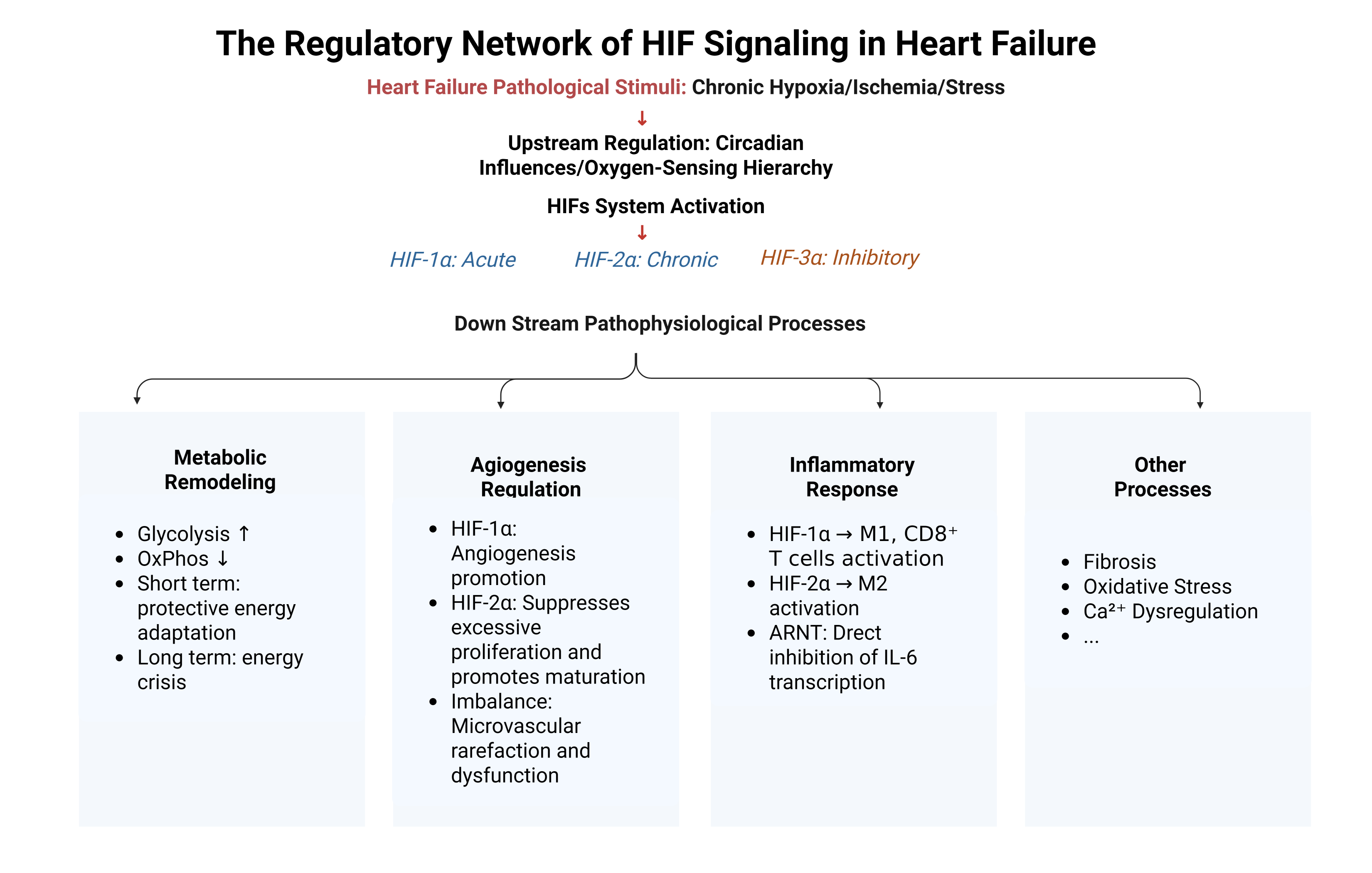

Supplement: Supplementary file 1 [file Image1.tif]
